# Yamanaka Factors in the Budding Tunicate *Botryllus schlosseri* Show a Shared Spatio-Temporal Expression Pattern in Chordates

**DOI:** 10.3389/fcell.2022.782722

**Published:** 2022-03-07

**Authors:** Virginia Vanni, Marika Salonna, Fabio Gasparini, Margherita Martini, Chiara Anselmi, Carmela Gissi, Lucia Manni

**Affiliations:** ^1^ Department of Biology, University of Padova, Padova, Italy; ^2^ Department of Biosciences, Biotechnologies and Biopharmaceutics, University of Bari “Aldo Moro”, Bari, Italy; ^3^ Stanford University, Hopkins Marine Station, Pacific Grove, CA, United States; ^4^ Institute for Stem Cell Biology and Regenerative Medicine, Stanford University School of Medicine, Stanford, CA, United States; ^5^ IBIOM, Institute of Biomembranes, Bioenergetics and Molecular Biotechnologies, Consiglio Nazionale Delle Ricerche, Bari, Italy; ^6^ CoNISMa, Consorzio Nazionale Interuniversitario per le Scienze Del Mare, Roma, Italy

**Keywords:** ascidians, asexual development, *Botryllus schlosseri*, *cMyc*, *Pou*, *Klf4*, *Sox2*, Yamanaka factors

## Abstract

In vertebrates, the four transcription factors *Sox2*, *c-Myc*, *Pou5f1* and *Klf4* are involved in the differentiation of several tissues during vertebrate embryogenesis; moreover, they are normally co-expressed in embryonic stem cells and play roles in pluripotency, self-renewal, and maintenance of the undifferentiated state in adult cells. The *in vitro* forced co-expression of these factors, named Yamanaka factors (YFs), induces pluripotency in human or mouse fibroblasts. *Botryllus schlosseri* is a colonial tunicate undergoing continuous stem cell-mediated asexual development, providing a valuable model system for the study of pluripotency in the closest living relatives of vertebrates. In this study, we identified *B. schlosseri* orthologs of human *Sox2* and *c-Myc* genes*,* as well as the closest homologs of the vertebrate-specific *Pou5f1* gene, through an in-depth evolutionary analysis of the YF gene families in tunicates and other deuterostomes. Then, we studied the expression of these genes during the asexual cycle of *B. schlosseri* using *in situ* hybridization in order to investigate their possible involvement in tissue differentiation and in pluripotency maintenance. Our results show a shared spatio-temporal expression pattern consistent with the reported functions of these genes in invertebrate and vertebrate embryogenesis. Moreover, *Myc*, *SoxB1* and *Pou3* were expressed in candidate stem cells residing in their niches, while *Pou2* was found expressed exclusively in the immature previtellogenic oocytes, both in gonads and circulating in the colonial vascular system. Our data suggest that *Myc*, *SoxB1* and *Pou3* may be individually involved in the differentiation of the same territories seen in other chordates, and that, together, they may play a role in stemness even in this colonial ascidian.

## Introduction

Stem cells (SCs) are undifferentiated cells capable of self-renewal and with the ability to differentiate into specialised cells. Given their pivotal role in animal development and regeneration, understanding the molecular mechanisms controlling their potency and evolution is of critical importance. Studies in SC biology are mainly based on mammals or other vertebrates, despite their limited regenerative capacities (Zhao et al., 2016). Tunicates are the sister group of vertebrates (Delsuc et al., 2006), and colonial tunicates are the only chordates able to regenerate entire organisms from circulating SCs (Rinkevich et al., 1995; Sunanaga et al., 2006; Voskoboynik et al., 2007; Brown et al., 2009; Kassmer et al., 2019; Manni et al., 2019; Scelzo et al., 2019). As such, this group offers unique opportunities to investigate the similarities and differences in SC biology between vertebrate and invertebrate chordates.

**FIGURE 1:**
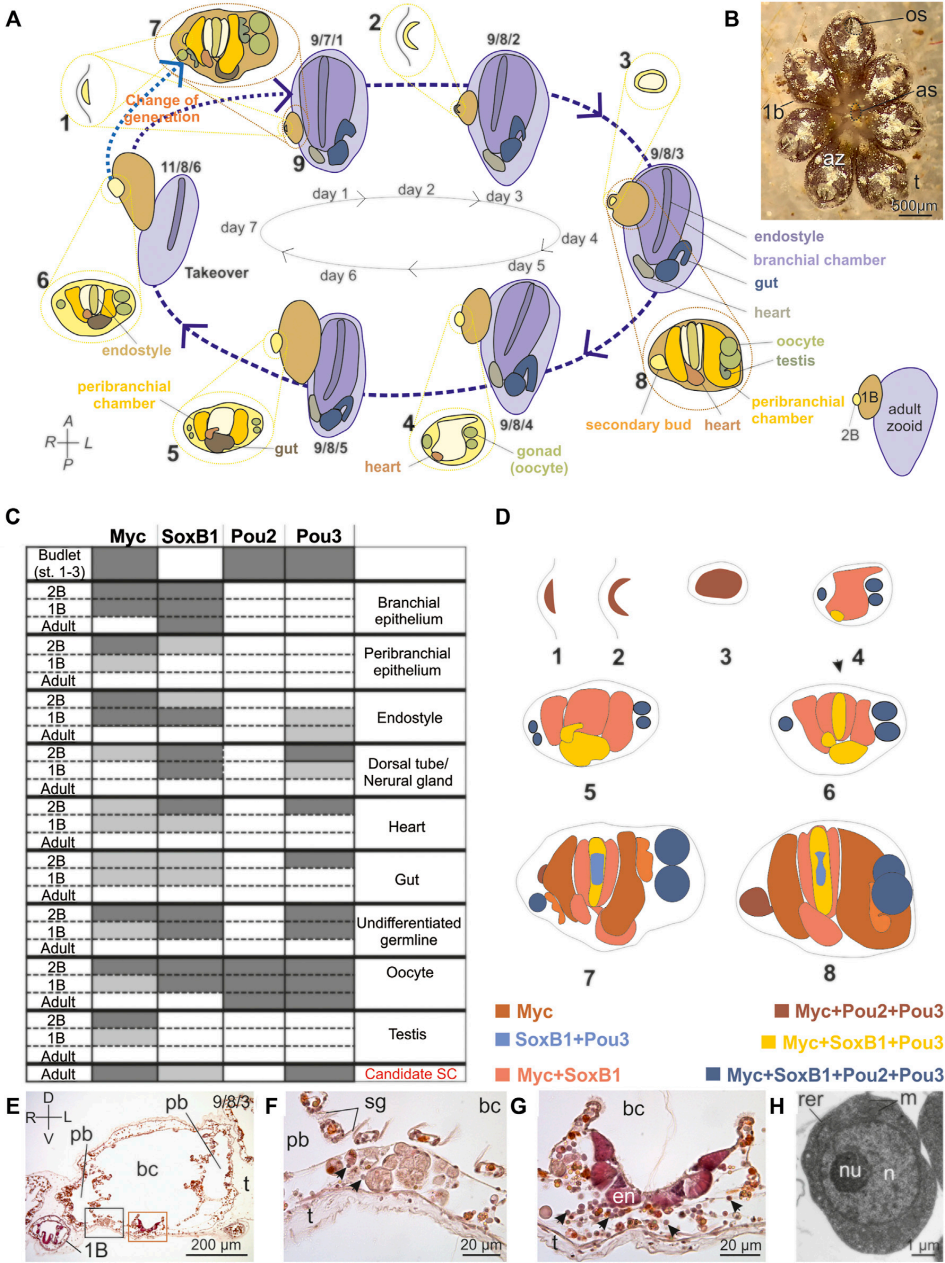
*Botryllus schlosseri* blastogenic cycle and Yamanaka Factors spatio-temporal expression pattern. **(A)** The weekly blastogenic cycle (dashed blue arrows) begins when a 2B emerges as a thickening of the parental peribranchial chamber epithelium accompanied by the epidermis (stage 1). The 2B then arches (stage 2) before it closes (stage 3) in a “double vesicle”: the outer vesicle is formed by the epidermis, the inner one by the original peribranchial epithelium. The inner vesicle undergoes invaginations (stage 4) to form the branchial and peribranchial chamber rudiments. In the lateral mesenchymal space between the inner and the outer vesicle germ cells reside whereas the heart rudiment forms postero-ventrally. The branchial chamber rudiment forms posteriorly the gut rudiment (stage 5), whereas on its ventral floor the endostyle becomes recognizable (stage 6). The passage to stage 7 (dotted light blue arrow) marks the change in generation when the 2B becomes a 1B, producing a new generation of 2Bs at stage 1. At stage 8, when the bud is at stage 2, the heart begins beating. In the following days (days 3–6), the 2B grows and differentiates organs. At the generation change, the 1B opens its siphons and becomes an adult (stage 9; dotted blue arrow). Concurrently, the previous generation of adult zooids undergoes resorption (takeover). Every week, at 18°C, there is a generation change and the lifespan of an individual (from 2B at stage 1 to adult zooid in regression) lasts ∼3 weeks. Numbers indicate the stages: numbers 1-6 refer to the 2B; numbers 7-8 to the 1B; numbers 9–11 to adult zooids. The triplet of numbers 9/7/1, 9/8/2, 9/8/3, 9/8/4, 9/8/5, and 11/8/6 refer to the colony phase; the first number referring to adult zooids, the middle to 1Bs and the last to 2Bs (Gasparini et al., 2015; Manni et al., 2007; Manni et al., 2014; Manni et al., 2019). 2Bs at stages 1–3 are in lateral view, other zooids are in ventral view. Colonies at the phases 9/7/1 and 9/8/2 are in early-cycle; colonies in 9/8/3–9/8/4 are in mid-cycle; colonies in 9/8/5–11/8/6 are in late cycle. Colour code refers to the different organs/anatomical parts. **(B)** Colony of *B. schlosser*i formed by seven adult zooids (az) grouped in a star-shaped system (dorsal view). 1 and 2Bs are hidden by adult individuals. Each adult zooid has its own oral siphon (os), whereas the cloacal siphons converge at the system center to open in a common atrial siphon (as). A transparent tunic (t) embeds the zooids. **(C)** Expression of *Myc*, *Soxb1*, *Pou2* and *Pou3* in the organs of 2Bs, 1Bs and adults based on ISH labelling data. Dark grey = strong labelling; light gray = weak labelling; white = absence of labelling. **(D)** Illustrations synthesizing *Myc*, *Soxb1*, *Pou2* and *Pou3* ISH labelling in 2 and 1Bs. Colour code refers to the different gene sets expressed in the different organs/anatomical parts, according to the colour of gene names. See **(A)** for anatomical details. The dorsal organ (in stage 7) and its derivative, i.e., the neural gland (in stage 8) are bordered by a dotted line and are found in the dorsal midline in 1Bs. Numbers below each illustration indicate the stages. **(E–G)** Transverse histological section **(E)** of an adult zooid with its right 1B. The triplet of numbers in the upper right corner indicates the colony phase. The black square area in **(E)** is enlarged in **(F)** to show a cell island. Note some cSCs, i.e., small round cells, about 5 µm in diameter, with high nucleus/cytoplasm ratio and without cytoplasmic specializations (arrowheads). They are close to large phagocytes. The brown square area in **(E)** is enlarged in **(G)** to show the endostyle niche, connective tissue rich in cSCs (arrowheads), ventral to the endostyle (en). bc: branchial chamber; pbe: peribranchial chamber; sg: stigmata; t: tunic. D–V: dorso-ventral axes; L–R: left-right axes. Hematoxylin-Eosin. **(H)** A cSC of the endostyle niche. m: mitochondria; n: nucleus; nu: nucleolus; rer: rough endoplasmic reticulum cistern. Transmission Electron Microscopy.

Among colonial tunicates, the ascidian *Botryllus schlosseri* is recognized as a model species for the study of SCs and asexual reproduction (Manni et al., 2007; Voskoboynik and Weissman, 2015; Manni et al., 2019). This species reproduces both sexually and asexually. In a colony, each individual (zooid) is hermaphroditic and produces swimming larvae via sexual reproduction. Larvae metamorphose into sessile individuals that reproduce asexually by budding (blastogenesis), forming clonal zooids grouped in star-shaped systems and embedded in a common tunic (Figures 1A,B). In each colony, three generations coexist: adult zooids, their primary buds (1Bs), and secondary buds (2Bs). In laboratory conditions (18°C), each week all adult zooids are resorbed during a process called takeover and replaced by their 1Bs; simultaneously, 2Bs become 1Bs and produce a new generation of 2Bs. Each new 2B originates from a thickening of the peribranchial epithelium of a 1B, which further develops into a vesicle undergoing organogenesis. Organogenesis nears completion when the 2B becomes a 1B where tissue differentiation occurs. *B. schlosseri* SCs are able to freely circulate in the colony haemocoel where, over generations, they contribute to tissue differentiation in buds (Kowarsky et al., 2021). Candidate SCs (cSCs) were identified in *B. schlosseri* as hemoblast, *i.e*., round cells, with a diameter of about 5 μm and a high nucleus-cytoplasm ratio (Rosental et al., 2018; Kowarsky et al., 2021). Two cSC transient niches have also been proposed in adult zooids (Figures 1E–H): the haemocoel, ventral to the anterior endostyle (the gland on the pharynx floor which produces the mucus net for filtration) (Voskoboynik et al., 2008; Rosental et al., 2018), and the “cell islands'', *i.e*., groups of haemocytes in the ventral body wall (Rinkevich et al., 2013). Cell islands are numerous in adult individuals belonging to early-cycle colonies and decrease in number toward the late-cycle. cSCs reside and proliferate in these transient niches and migrate before takeover through the haemocoel to relocate in newly formed niches of the following generations (Rinkevich et al., 2013).

The four transcription factors *c-Myc, Sox2*, *Pou5f1* (also named *Oct3/4*) and *Klf4* are of interest given their role in tissue differentiation and in both regulating and inducing pluripotency in vertebrates. *Sox2* and *Pou5f1* are involved in maintaining pluripotency of embryonic stem cells (Niwa et al., 2000; Avilion et al., 2003; Boyer et al., 2005), while *c-Myc* and *Klf4* play a key role in self-renewal and maintenance of the undifferentiated state (Cartwright et al., 2005; Li et al., 2005). Remarkably, the forced co-expression of these four transcription factors, also named Yamanaka Factors (YFs), is sufficient to reprogram murine and human fibroblasts into induced pluripotent stem (iPS) cells (Takahashi and Yamanaka, 2006; Takahashi et al., 2007).

*c-Myc* belongs to the MYC gene family, characterized by a C-terminal basic-helix-loop-helix-leucine zipper (bHLHZ) domain mediating protein dimerization and DNA binding, and two N-terminal *trans*-activation motifs (Atchley and Fitch, 1995; James and Eisenman, 2002). Vertebrates possess multiple *Myc* genes (*c-Myc*, *N-Myc*, *L-Myc*), with *c-Myc* involved in cell division and growth control, and considered a central hub for pluripotent SC regulatory networks (Chappell and Dalton, 2013; Fagnocchi and Zippo, 2017). In contrast, two genes are found in a cnidarian with high regenerative ability (Hartl et al., 2010), and only a single homolog is present in *Drosophila* and most other invertebrates (Gallant, 2006; McFerrin and Atchley, 2011). Despite differences in gene number, the expression of the single *Myc* gene in the undifferentiated cells of invertebrates suggests that its function is similar to that of *c-Myc* in vertebrates (Kawamura et al., 2008; Hartl et al., 2010; Fujiwara et al., 2011).

*Sox2* is a member of the ancient SOX gene family that has undergone frequent gene duplications and is therefore divided into ten subgroups (A-J) based on the similarity of the DNA-binding domain (Wegner, 1999; Bowles et al., 2000). In vertebrates, *Sox2, Sox1* and *Sox3* comprises the SoxB1 group, while the close SoxB2 group consists of *Sox14* and *Sox21*. Invertebrates have only a single member each of the SoxB1 and SoxB2 groups (Bowles et al., 2000; Heenan et al., 2016). The role of *SoxB1* in pluripotency appears to be conserved among invertebrates (Onal et al., 2012; Niwa et al., 2016), where it is fundamental for neural development and regeneration (Mizuseki et al., 1998; Buescher et al., 2002; Bylund et al., 2003; Graham et al., 2003; Miya and Nishida, 2003; Meulemans and Bronner-Fraser, 2007; Marlow et al., 2009; Ross et al., 2018) and is expressed in multiple adult self-renewing epithelia (Arnold et al., 2011).

*Pou5f1* belongs to the metazoan POU gene family, characterized by a modular DNA-binding domain (POU) consisting of a N-terminal POU-specific domain (POUs) and a C-terminal homeodomain (POUh) separated by a linker of variable length. Based upon similarity across the entire POU domain, *Pou* genes are grouped into six classes (Rosenfeld, 1991), that have evolved through multiple lineage-specific events of gene duplications and losses (Frankenberg and Renfree, 2013; Gold et al., 2014; Onichtchouk, 2016). *Pou5f1* belongs to the vertebrate-specific POU5 class (also including *Pou5f3* and the primate/rodent-specific *Pou5f2*), hypothesised to be arisen from a Pou3-like ancestor together with the bilaterian-specific POU2 class (Gold et al., 2014).

*Klf4* is a member of the Kruppel like factors (*Klf*) family, characterized by the presence of three C-terminal highly conserved zinc finger motifs and a high diversification of the associated transactivation/repression domains (Vangapandu and Ai, 2009; Presnell et al., 2015). Depending on their structure and/or function, the *Klf* members are partitioned into different groups whose number varies across studies (McConnell and Yang, 2010; Pei and Grishin, 2015; Presnell et al., 2015; Pollak et al., 2018). Pei and Grishin (2015) found a minimum of 18 *Klf* genes in vertebrates and classified them into seven groups (A-G), with invertebrate deuterostomes possessing a single member of each group and the loss of *KlfE* in the ascidian *Ciona intestinalis*. Together with *Klf1/2/17,* the human *Klf4* belongs to the group *KlfA.*

In this study we investigate the presence and the evolution of the YF genes in ascidians, and analyze when and where the orthologs of the vertebrate YF genes are expressed in the blastogenetic cycle of *B. schlosseri* by using *in situ* hybridization (ISH) experiments. Our results show patterns of expression consistent with the reported function of these genes in invertebrate and vertebrate development and differentiation. Moreover, the expression of *Myc*, *SoxB1* and *Pou3* is retrieved in cSCs residing in cSC niches, suggesting these transcription factors may play a role in the maintenance of undifferentiated state, and highlighting the necessity to better investigate whether similar molecular mechanisms control pluripotency in vertebrates and tunicates.

## Methods

### Identification and Evolution of Yamanaka Factors in Chordates

The *in silico* identification of ascidian YF genes and the evolutionary analyses of the YF gene families in chordates were performed on the species listed in Supplementary Table 1, as detailed in Supplementary Data Sheet 1.

### *B. schlosseri* Colonies Rearing

*B. schlosseri* colonies were collected in the Venice Lagoon and reared at the Department of Biology (University of Padova) in standard laboratory condition (Sabbadin, 1955; Sabbadin, 1960). Colonies were observed daily and fixed at 6 developmental phases as defined by the staging method developed by Sabbadin (1955) (Figure 1A).

### RNA Extraction, Yamanaka Factors cDNA Sequencing, and *in Situ* Hybridization

In order to validate the *B. schloss*eri predictions of *SoxB1*, *Myc*, *Pou2* and *Pou3*, and to develop probes for ISH experiments, total RNA was extracted from *B. schlosseri* single colonies according to the protocol of Campagna et al. (2016), followed by cDNA synthesis, cloning and sequencing. Whole-mount ISHs were carried out as described in Franchi and Ballarin (2014) using at least three samples per stage. Detailed protocols are in Supplementary Data Sheet 1.

### Histology and Transmission Electron Microscopy

Samples have been treated according to the classical protocols for histology (Hematoxylin-Eosin) and Transmission Electron Microscopy (TEM); details on the protocols are reported in Supplementary Data Sheet 1.

## Results

### Annotation of the YF Gene Families in Ascidians

A total of 47 coding sequences (CDS) of the YF families were re-annotated or annotated *ex novo* in the reference genomes of the 11 analysed ascidians (see genes with “_p” suffix in Supplementary Tables 2–5, Fasta sequences in Supplementary Data Sheet 2–5, and details in Supplementary Data Sheet 1). Original misannotations, causing partial or anomalous CDS identification, were mainly due to: failure in exon identification, erroneous exon boundaries, incorrect assignment of exons to genes, assembly anomalies, or sequencing errors.

### A Single Ancestral *Myc* Gene Is Expressed Extensively During Bud Development

**FIGURE 2:**
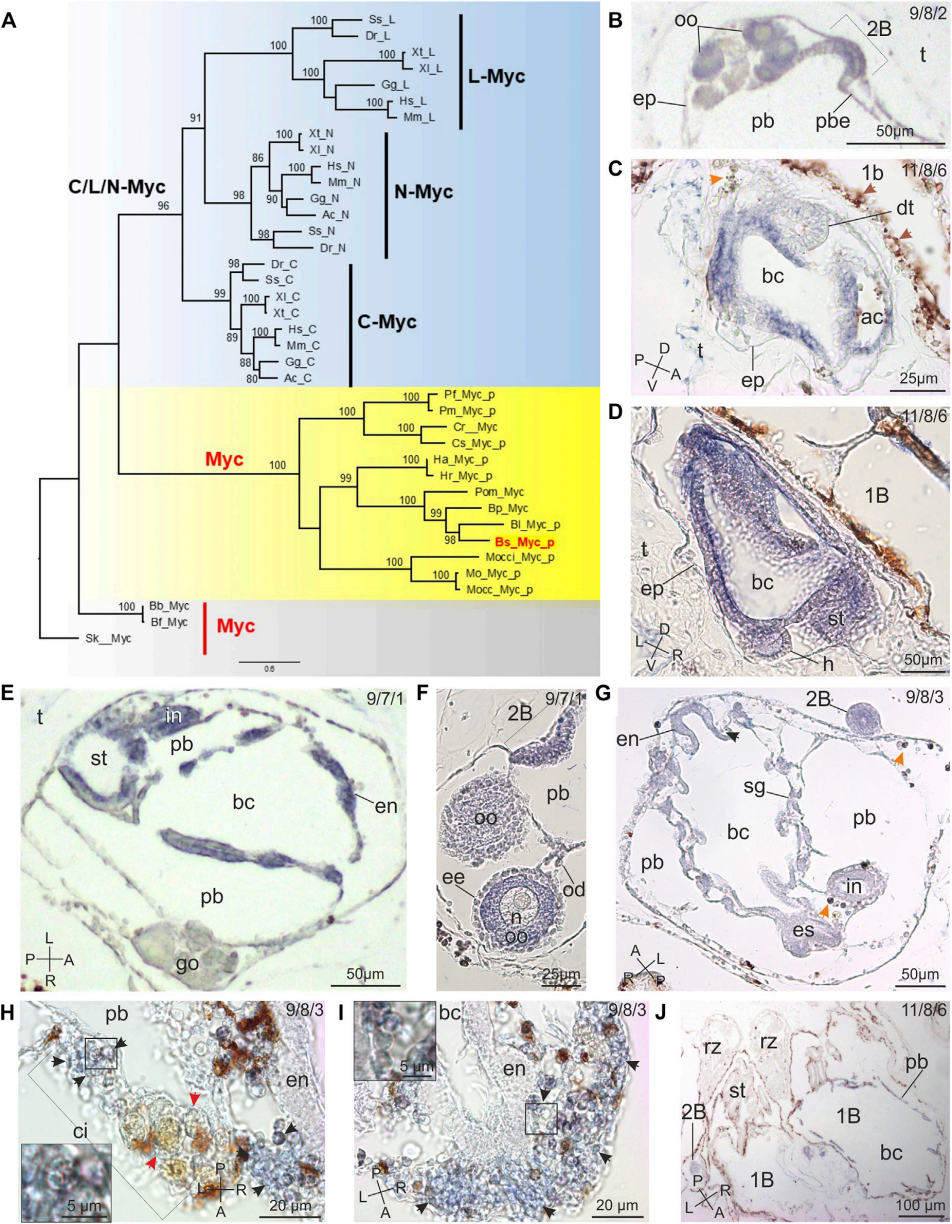
*Myc* evolution and expression pattern in *B. schlosseri*. **(A)** ML evolutionary tree of the MYC family (978 amino acid sites; best-fit substitution model JTT+G+I+F; bootstrap on 100 replicates; *Myc* of the echinoderm *Strongylocentrotus purpuratus* used as outgroup). Only bootstrap values ≥70% are shown near each node. Red: *B. schlosseri* sequence used in ISH; yellow: ascidians; light blue: vertebrates; grey: other deuterostomes. Sequence data are in Supplementary Table 2. Sequences with the "_p" suffix were annotated *ex novo* or re-annotated in this study. Species classification is reported in Supplementary Table 1, and species abbreviations are: Ac: *Anolis carolinensis*; Am: *Alligator mississippiensis*; Bb: *Branchiostoma belcheri*; Bf: *Branchiostoma floridae*; Bl: *Botrylloides leachii*; Bpm: *Botryllus primigenus;* Bs: *Botryllus schlosseri;* Btub: *Botryllus tuberatus;* Cm: *Callorhinchus milii;* Cr: *Ciona robusta;* Cs: *Ciona savignyi;* Dr: *Danio rerio;* Gg: *Gallus gallus;* Ha: *Halocynthia aurantium;* Herd: *Herdmania curvata;* Hr: *Halocynthia roretzi;* Hs: *Homo sapiens;* Mm: *Mus musculus;* Mo: *Molgula oculata;* Mocc: *Molgula occulta;* Mocci: *Molgula occidentalis;* Pf: *Phallusia fumigata;* Pm: *Phallusia mammillata;* Pom: *Polyandrocarpa misakiensis;* Scla: *Styela clava;* Sk: *Saccoglossus kowalevskii;* Sp: *Strongylocentrotus purpuratus;* Ss: *Salmo salar;* Xl: *Xenopus laevis;* Xt: *Xenopus tropicalis*. **(B–J)** Myc spatiotemporal expression during blastogenesis on sections of colonies processed for ISH. The triplet of numbers in the upper right corner indicates the colony phase. A–P: antero-posterior axes; D–V: dorso-ventral axes; L–R: left-right axes. **(B)** Detail of an early 1B with its 2B (stage 2) in the form of arch of thickened epithelium (2b). Note the gonad rudiment in the mantle. ep: epidermis; oo: oocytes; pb: peribranchial chamber in 1B; pbe: peribranchial chamber epithelium; t: tunic. **(C,D)** Two sagittal sections of a 2B before the generation change (stage 6) close to the parental 1B (1b). Note that the forming branchial (bc) and atrial (ac) chambers, the stomach (st) and the heart (h) are clearly marked. In **(C)**: brown arrows: non-labelled vacuolated cells with brown precipitates; orange arrow: non-specific labelling in an immunocyte (morula cell). dt: dorsal tube; ep: epidermis; t: tunic. **(E,F)** Two sections of 1Bs just after the generation change (stage 7; frontal section in **(E)**). In the 1B, epithelia of branchial (bc) and peribranchial (pb) chambers, endostyle (en), stomach (st), and intestine (in) are positive to the *Myc* probe. In **(F)**, the labelled 2B (stage 1) is also recognizable close to the gonad (go); note that the oocyte (oo) cytoplasm is marked but the follicle envelopes (ee), the oocyte nucleus (n), and the oviduct (od) are not. t: tunic. **(G)** Frontal section of an advanced 1B (stage 8) with its 2B (stage 3; 2b). Several tissues are labelled in 1B: endostyle (en), branchial (bc) and peribranchial (pb) chamber epithelia, endostyle (en), esophagus (es), and intestine (in). The 2B is also marked. Black arrow: labelled hemoblast; orange arrows: non-specific labelling in immunocytes (morula cells). **(H,I)** Details of cell island (ci) and endostyle (en) in filter feeding adult zooids. Note that cSCs (enlarged in insets) are marked (arrowheads); the large, brown cells in cell island are not-labelled phagocytes (red arrows in **(H)**). bc: branchial chamber; pcb: peribranchial chamber. **(J)** Section of regressing zooids (rz) close to large 1Bs (1b) of a colony in takeover. Note that there is no labelling in regressing zooids, whereas branchial (bc) and peribranchial (pb) chamber epithelia and endostyle in 1B, and the 2B (2b) are marked. st: stomach.

A single *Myc* gene was present in *B. schlosseri* and in all the other ascidians and invertebrate deuterostomes analyzed, and our evolutionary tree clearly shows that this single gene gave rise to the three vertebrate paralogs (*L-Myc*, *N-Myc* and *C-Myc*) through two duplication rounds (Figure 2A). Noteworthy, the evolutionary history of each paralog corresponds exactly with the species tree. Indeed the ascidian sequences cluster according to taxonomic classification in orders and families with high statistical support (Supplementary Table 1 and bootstrap values in Figure 2A).

The *Myc* gene structure is highly conserved in ascidians and consists of two coding exons separated by a very long intron likely responsible for the incorrect *Myc* annotation in all ascidians but *Ciona robusta* (range: 10.1–23.8 kb in all analysed ascidians except for the 3–4.6 kb of Molgulidae; see Supplementary Table 2 and Supplementary Data Sheet 1). Remarkably, in all ascidians the second exon encodes for both the bHLHZ domain and a long C-terminal region (mean length and standard deviation: 118 ± 31 amino acids), which are absent in all other analyzed vertebrates and invertebrates genes.

**FIGURE 3:**
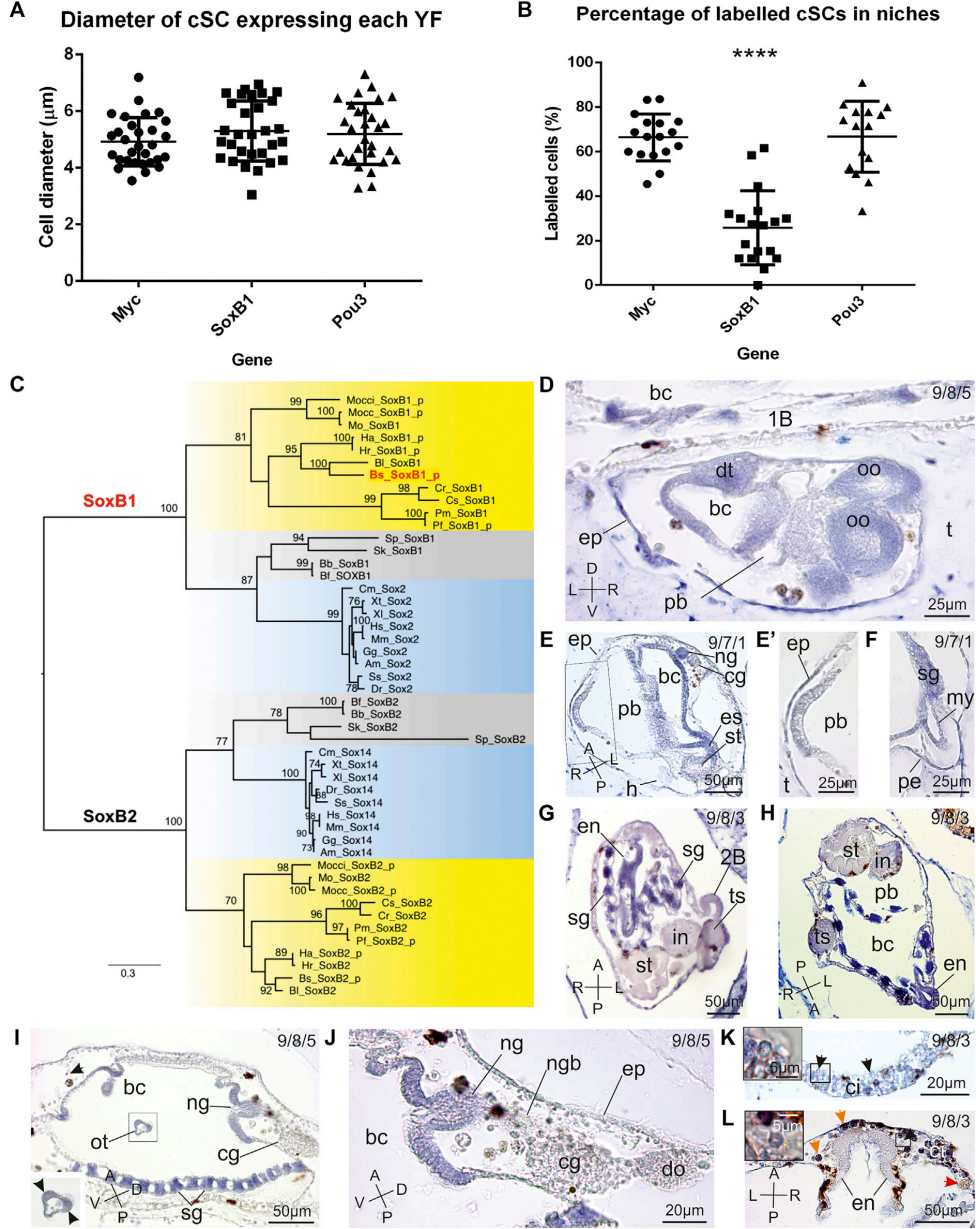
Morphometrics analyses of labelled candidate SCs in niches, *SoxB* evolution and *SoxB1* expression pattern in *B. schlosseri*. **(A)** Histogram showing the diameter of cSCs found labelled in niches. Statistical tests show no significant difference among the diameter of cells expressing *Myc*, *SoxB1* and *Pou3*. **(B)** Histogram showing the percentage of cSCs found labelled in niches. Candidate SCs found positive for the expression of *SoxB1* are significantly less numerous than cSCs expressing *Myc* and *Pou3* (**** = *p*-value<0.0001, ANOVA test), but the high percentage of cells expressing these last two genes suggest a coexpression in at least a part of cSCs. **(C)** ML evolutionary tree of the *SoxB* subgroup (524 amino acid sites; best-fit substitution model JTT+G+I+F; bootstrap on 100 replicates; *SoxB2* cluster used as outgroup). Only bootstrap values ≥70% are shown near each node. Red: *B. schlosseri* sequence used in ISH; yellow: ascidians; light blue: vertebrates; grey: other deuterostomes. Species abbreviations are as in Figure 2. Species classification and sequence data are in Supplementary Tables 1, 3, respectively. Sequences with the "_p" suffix were annotated *ex novo* or re-annotated in this study. **(D–L)** *SoxB1* spatio-temporal expression pattern during blastogenesis on sections of colonies processed for ISH. The triplet of numbers in the upper right corner indicates the colony phase. A–P: antero-posteriore axes; D–V: dorso-ventral axes; L–R: left-right axes. **(D)** Sagittal section of an advanced 2B (stage 5). Branchial (bc) and peribranchial (pb) chamber epithelia, dorsal tube (dt), and oocytes (oo) are labelled. 1b: parental 1B; ep: epidermis; t: tunic. **(E,F)** Sagittal-oblique section of a 1B just after the generation change (stage 7), with its 2B (stage 1) (square area in **(E)** is enlarged in E′). The 2B is not labelled; while several tissues in the 1B (branchial chamber (bc) epithelium, neural gland aperture (ng), esophagus (es), and stomach (st)) are marked. **(F)** shows a detail of the heart, in which both the pericardium (pe) and the myocardium (my) are labelled. cg: cerebral ganglion; ep: epidermis; sg: stigma; t: tunic. **(G,H)** Two frontal sections of advanced 1Bs (stage 8). Stigmata rudiments (sg) and the endostyle (en) are strongly marked; the gut is weakly marked. Both the female and male gonads are strongly labelled 2b: left 2B; bc: branchial chamber; in: intestine; pb: peribranchial chamber; st: stomach; ts: testis. **(I,J)** Oblique section of a 1B approaching the generation change in **(I)** (stage 8) and detail of the neural complex in **(J)**. The stigmata (st) and the aperture of the neural gland (ng) are strongly marked. Note that the oral tentacle (ot) is also labeled at the coronal sensory cell level (arrowheads in inset), whereas the cerebral ganglion (cg) and close hemocytes are not marked. bc: branchial chamber; do: dorsal organ; ep: epidermis; ngb: neural gland body; Black arrow in **(I)**: labelled hemoblast. **(K,L)** Detail of SC niches in adult zooids: cell islands (ci) and the subendostyle niche. Note some cSCs (enlarged in insets) marked by *SoxB1 (arrowheads)*. Orange arrows: non-specific labelling in immunocytes (morula cells); red arrow: non-labelled phagocytes; en: endostyle.

In the blastogenetic cycle, ISHs demonstrated that *Myc* was expressed in all the tissues across all developmental stages except in the nervous system rudiment both in 2Bs, from the early stages of development, and in 1Bs (Figures 1C,D, 2). *Myc* expression was weak as buds progressed in development, disappearing in adult tissues, with the exception of cells showing a morphology comparable to cSCs (round cells, with a diameter of 4.92 ± 0.85 µm and a high nucleus-cytoplasm ratio) (Figures 2H,I, 3A,B, Supplementary Data Sheet 6), aggregated in the ventral region in the proximity of the endostyle, and in cell islands in all the phases. These cells were the only labelled hemocytes (Ballarin and Cima, 2005): immunocytes (Figures 2C,G,H) exhibited non-specific labelling, whereas phagocytes (Figure 2H) and vacuolated cells (Figures 2C,D) were never labelled.

### *SoxB1* Is Amply Expressed During Bud Development but Persists in Adult Zooids Only in Candidate Stem Cells

In order to unambiguously identify the ascidian orthologs of the human *Sox2* gene, we reconstructed the evolutionary history of both the *SoxB1* and *SoxB2* groups in deuterostomes. Our tree clearly delineates the two groups (Figure 3C) and shows a general agreement between the gene tree and the ascidian species tree (see taxonomy classification in Supplementary Table 1), although not all nodes have statistically significant bootstrap values (Figure 3C). Both *SoxB1* and *SoxB2* have conserved gene structure in ascidians (with 2 and 4 coding exons, respectively), and were re-annotated in *B. schlosseri* and in several other ascidians (Supplementary Table 3 and Supplementary Data Sheet 1).

*SoxB1* expression was absent in early stages of blastogenesis until the late 2B stages (stages 4-5–6). Here, *SoxB1* transcript was found in the developing branchial epithelium, oesophagus, heart and neural complex (Figures 1C,D, 3). In 1Bs, the gene was additionally expressed in stigmata and endostyle. In adult zooids in early-, mid-, and late-cycle, the expression was restricted to the stigmata and to a few cSCs (round cells, with a diameter of 5.3 ± 1.06 µm and a high nucleus-cytoplasm ratio; Figure 3A; Supplementary Data Sheet 6) aggregated in the region ventral to the endostyle and in cell islands. Among hemocytes, these cells were the only positive ones during the blastogenetic cycle (Figure 3L). However, cSCs expressing *SoxB1* were less numerous than those expressing the other YFs, probably in relationship with their functional state (Figure 3B, Supplementary Data Sheet 6).

### *Pou2* Expression Is Female Gonad-Restricted Whereas *Pou3* has a Wider Expression Pattern

**FIGURE 4:**
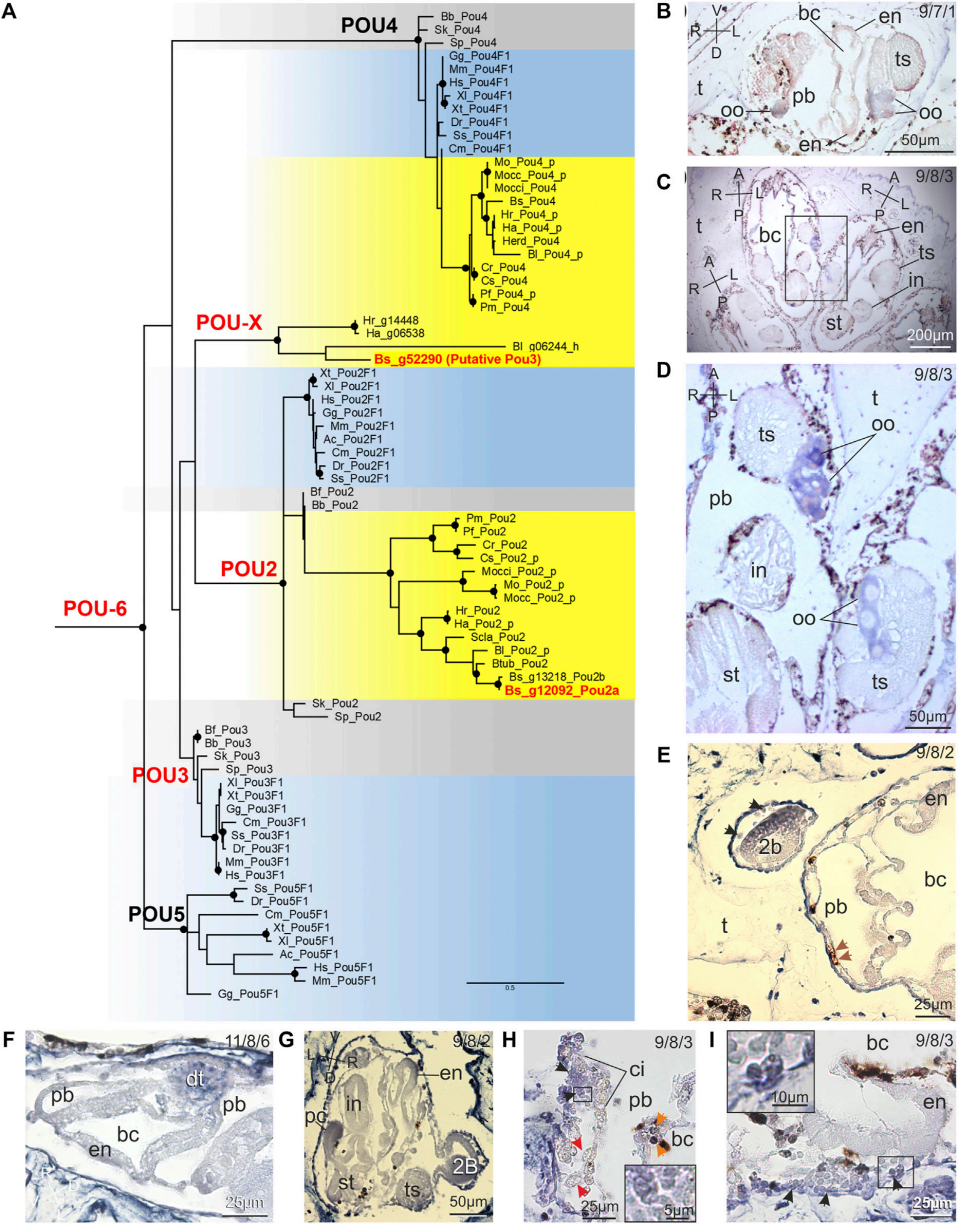
Pou evolution and expression pattern of *Pou2* and *Pou3* in *B. schlosseri*. **(A)** ML evolutionary tree of the POU family based on the C-terminal region (199 amino acid sites; best-fit substitution model JTT+I+G; bootstrap on 100 replicates; *Pou6* used as outgroup). The cladogram of the full ML tree, containing all bootstrap values and all *Pou6* outgroups, is shown in Supplementary Image 1. Black dots: bootstrap values ≥70%; red: *B. schlosseri* sequences used in ISH; yellow: ascidians; light blue: vertebrates; grey: other deuterostomes. Species abbreviations are as in Figure 2. Species classification and sequence data are in Supplementary Table 1 and in Supplementary Table 4, respectively. Sequences with the “_p” suffix were annotated *ex novo* or re-annotated in this study; the sequence with “_h” suffix is a hybrid between a genomic and a mRNA sequence (see Supplementary Table 2). **(B–I)** *Pou2* **(B,D)** and *Pou3* **(E–I)** spatio-temporal expression during blastogenesis on sections of colonies processed for ISH. The triplet of numbers in the upper right corner indicates the colony phase. A–P: antero-posteriore axes; D–V: dorso-ventral axes; L–R: left-right axes. **(B)** Frontal section of early 1B (stage 8). Only the female gonad exhibits labelling. bc: branchial chamber; en: endostyle; oo: oocyte; pb: peribranchial chamber; t: tunic; ts: testis. **(C,D)** Adult zooids (frontal section). Only the oocytes (oo) in the female gonad exhibit labelling. The square area in **(C)** is enlarged in **(D)**. bc: branchial chamber; en: endostyle; in: intestine; pb: peribranchial chamber; st: stomach; t: tunic; ts: testis. **(E)** A strongly labelled early 2B (stage 2) close to its parental 1B (stage 8), in which the endostyle (en) is weakly marked. Brown arrows: non-labelled vacuolated cells; black arrows: labelled hemoblasts. bc: branchial chamber; pb: peribranchial chamber; t: tunic. Late 2B (stage 6), just before the generation change. The dorsal tube shows strong labelling, whereas the other tissues do not. bc: branchial chamber; en: endostyle; pb: peribranchial chamber. **(G)** 1B (stage 8) with its early 2B (stage 2). The endostyle (en) and the pyloric caecum (pc) of the 1B are weakly labelled, whereas the 2B is strongly marked. in: intestine; st: stomach; ts: testis. **(H, I)** Details of a cell island (ci) and the endostyle (en) of an adult individual (stage 9). Candidate SCs (enlarged in insets) are positive to the probe (arrowheads). In **(H)**, orange arrows: non-specific labelling in immunocytes (morula cells); red arrows: non-labelled phagocytes. bc: branchial chamber; pb: peribranchial chamber.

Due to its complexity, the evolutionary history of the POU family was reconstructed including the POU2, POU3, POU4 and POU5 classes, with vertebrate representatives of the POU6 class as outgroups (Figure 4 and Supplementary Image 1). In the reconstructed tree all basal nodes remain unresolved and a polytomous POU3 “bush” can be observed (Figure 4A). Increasing the number of replicates does not improve the support of these nodes, indeed bootstopping tests did not converge even after 1,000 trees. However, five highly supported monophyletic clades are identified (see nodes with dots in Figure 4A), corresponding to: 1) the POU2 class; 2) the POU4 class; 3) the vertebrate-specific POU5 class; 4) all analysed vertebrate *Pou3f1* genes; and 5) four fast-evolving ascidian sequences (POU-X clade in Figure 4A). Thus our tree confirms the absence of POU5 in ascidians and allows the unambiguous identification of the ascidian orthologs of POU2 and POU4 classes, remarkably present in all 11 analysed ascidian genomes (Figure 4A). Moreover, the topology of both the POU2 and POU4 ascidian clades corresponds to the expected species tree based on the ascidian classification, and a nearly conserved intron/exon structure is observed within each of these clades (see column “Ascidian Classification” and “N° of coding exons” in Supplementary Table 4).

Since a published phylogenetic reconstruction performed on all metazoans and all POU classes hypothesized a Pou3-like gene as the last common ancestor of the POU5/3/2 polytomous clade (Gold et al., 2014), we focused our expression pattern analyses on *Pou2* and *Pou3* (see *Discussion*).

Two *Pou2* genes, named *Pou2a* and *Pou2b*, have been identified in the *B. schlosseri* genome (Bs_g12092_Pou2a and Bs_g13218_Pou2b in Figure 4A). They differ only for the first and last exon while the remaining intron/exon structure is identical (10 exons, with 16 over a total of 18 intron/exon boundaries falling in exactly the same protein position). Thus, the encoded proteins are nearly identical (97% amino acid identity over 255 alignable sites) but have short and completely different N- and C-terminal ends, and different total lengths (359 for *Pou2a vs.* 293 amino acids for *Pou2b*). Since the C-terminal region of *Pou2a* is more similar than *Pou2b* to the homologous regions of the closest species *B. leachii,* only *Pou2a* was experimentally validated (Supplementary Data Sheet 1) and used in ISH experiments. Indeed, we hypothesize that *Pou2b* is the result of errors in genome assembly as the entire mapping regions and even the intron sequences of *Pou2a* and *Pou2b* are almost identical (94% nucleotide identity with 2% gap over 4.6 kb). However, a very recent gene duplication cannot be ruled out.

The POU-X clade only includes representatives of the families Styelidae and Pyuridae and was putatively assigned to the POU3 class based on two considerations: the single-exon gene structure, typical of all other *Pou3* of deuterostomes here analysed (Supplementary Table 4), and the presence of elements known to impair the tree resolution (*i.e.*, the very long branches of POU-X sequences associated with the intrinsic polytomy of the POU3 class). Therefore, the Bs_g52290 sequence of POU-X clade (Figure 4A) was considered a valid *B. schlosseri Pou3* and used in subsequent ISH experiments.

*Pou2* was found expressed in the female germline and, more specifically, in oocytes in previtellogenesis in 1Bs, 2Bs, adult zooids, and circulating in the haemocoel (Figures 1C,D, 4A–D). Moreover, its transcript was retrieved in cells of early developing 2B. *Pou3* was found expressed in early-cycle of blastogenic development (Figures 1C,D, 4E–I). Subsequently its expression was restricted to the germline and the rudiments of the neural complex, heart, and digestive system. In the following phases of bud development, the expression persisted at high levels only in the female and male germlines, while lower levels were found in the nervous system. In adult zooids, the presence of *Pou3* transcript persisted in germline and in cSCs (round cells, with a diameter of 5.2 ± 1.08 µm and a high nucleus-cytoplasm ratio; Figure 3A, Supplementary Data Sheet 6) found near the endostyle and in cell islands in all the phases. Moreover, it could be retrieved in the endostyle, with an antero-posterior pattern of respectively high and low expression levels. As reported for *Myc* and *SoxB1*, cSCs were the only hemocytes expressing *Pou3* during the blastogenetic cycle (Figure 4H).

### The *Klf* Gene Family

The KLF proteins are characterized by a high N-terminal sequence variability that makes their alignment unreliable except in the three zinc finger motifs. The reconstructed evolutionary tree, based on the 93 reliably alignable sites of the zinc finger domain and including the *KlfA/B/C/D/F/G* groups, is almost completely unresolved (Supplementary Image 2) and shows a mildly supported *KlfA* clade (75% bootstrap) inside a wide polytomous *KlfA/B/C/D* clade. Only a partial *KlfA* sequence, corresponding only to the Zn finger domain, was found and annotated by us in an earlier assembly version of the *B. schlosseri* genome (see “Notes” in Supplementary Table 5), suggesting that the *KlfA* absence in the current genome version was due to assembly problems. The very high conservation among groups of the available *KlfA* region (*i.e.*, the Zn finger domain) prevented us from designing target-specific ISH probes able to discriminate between the different *Klf* groups, therefore no gene expression analyses were performed on this gene.

### Yamanaka Factors Are Individually Expressed in Several Organ Rudiments of the Early Bud and, Except *Pou2*, in Candidate Adult Stem Cells in Proposed Niches

An overview of the spatio-temporal expression pattern of *Myc*, *SoxB1*, *Pou2* and *Pou3* during the blastogenic cycle is summarised in Figures 1C,D. The endostyle, neural complex, heart, gut, undifferentiated germline, and female germline all expressed three factors during the same developmental stages: *Myc*, *SoxB1* and *Pou3*. The same factors were expressed during the entire cycle in some cSCs, showing comparable dimensions among samples (Figure 3A), in both the SC niches of adult individuals and in hemocele. Almost 70% of adult cSCs in niches expressed *Myc* and *Pou3* (Figures 3A,B, Supplementary Data Sheet 6), suggesting that these two factors should be coexpressed in at least 20% of cSCs. However, SoxB1 is expressed in a lower percentage of cells (Figures 3A,B, Supplementary Data Sheet 6). Of note is that only the female gonad additionally expressed *Pou2*. *Myc* and *Pou* (both *Pou2* and *Pou3*) *g*enes were expressed in early 2Bs. *Myc* and *SoxB1* were recognised during the development of the branchial and peribranchial epithelia, whereas *SoxB1* and *Pou3* were present during brain formation. Finally, the testis expressed only *Myc,* and only during development, whereas the adult stigmata maintain the expression of *SoxB1*.

## Discussion

All YF gene families have a complex evolutionary history characterized by several gene duplications, some of which also occurred in the ancestor of vertebrates. Our evolutionary analyses show that a one-to-many relationship exists between ascidian and human orthologs of some YF genes, which means that the ascidian gene has more than one ortholog in humans. Indeed, the ascidian *SoxB1* is the ortholog not only of the human/vertebrate YF *Sox2*, but also of *Sox1* and *Sox3*. Likewise, the ascidian *Myc* gene is the ortholog not only of the human/vertebrate YF *c-Myc*, but also of *N-Myc* and *L-Myc*. This implies that the functional role and the expression pattern of these ascidian genes could have been inherited by one or many human/vertebrate orthologs. With that in mind, we have analysed the expression pattern of the YF genes in *B. schlosseri*, a highly regenerative model species belonging to the taxon evolutionarily closest to vertebrates.

Our results show that *Myc* is expressed in all developing tissues of buds and in cSCs in niches (Figures 1C,D), suggesting a crucial role in undifferentiated territories. Noteworthy, *Myc* expression in cSCs in their niches is a new finding, not observed in previous studies on *Myc* expression in two other colonial ascidians. Indeed, in *Botryllus primigenus*, *Myc* was proposed as a marker of undifferentiated, but not proliferative, somatic and germline SCs, as it was expressed by multipotent epithelial cells in buds, undifferentiated coelomic cells, and developing gonads (Kawamura et al., 2008). In *Polyandrocarpa misakiensis, Myc* expression was observed in the atrial epithelium in the organ-forming region of developing bud, and in fibroblast-like cells, which participate in the organogenesis together with the epithelial cells (Fujiwara et al., 2011). Moreover, the noteworthy identification of an ascidian-specific C-terminal tail, located just downstream the bHLHZ domain, incentivises further investigation on the role and the possible additional functions performed by this protein region.

As summarized in Figure 1C, *SoxB1* is widely expressed during *B. schlosseri* asexual development. It is found in the developing nervous system, as has also been reported for *Sox2* and other SoxB1 group genes in many metazoans (Wood and Episkopou, 1999; Penzel et al., 1997; Rex et al., 1997; Meulemans and Bronner-Fraser, 2007; Ferrero et al., 2014; Ross et al., 2018). In the developing nervous system we also detected the expression of *Pou3* (Figure 1C), known to be involved in neural system development in vertebrates and other invertebrates, (Candiani et al., 2002; Cole and Arnone, 2009; Wollesen et al., 2014; Garner et al., 2016; Wei et al., 2016; Prünster et al., 2019). The expression, in samples at the same developmental stages, of *SoxB1* and *Pou3* in the developing nervous system, observed here and in previous studies (Iwafuchi-Doi et al., 2011; Hui et al., 2015), suggests a co-regulation of these two factors during neurogenesis. We also retrieved *SoxB1* expression in the developing oesophagus, stomach and proximal intestine, suggesting a role in the differentiation and maintenance of these epithelia. This result corresponds with the *Sox2* expression in the adult stomach tissues of mammals (Arnold et al., 2011). Moreover, *SoxB1* was found expressed in the endostyle of 1B, and in cSCs of the endostyle niche and cell islands, indicating potential involvement in stemness. Supporting this hypothesis, Niwa et al. (2016) demonstrated that *C. robusta* and other invertebrate *SoxB1* proteins are able to replace the pluripotency-associated function of mouse *Sox2.* Thus the role of *SoxB1* in the control of pluripotency in *B. schlosseri* is plausible.

Our evolutionary analyses demonstrate the absence of the POU5 class in ascidians, indicating that POU5 arose after the tunicate-vertebrate divergence. Since there is no true ortholog of *Pou5f1* to be investigated in *B. schlosseri*, the ancestor of the POU5 class could instead be investigated in its place. Indeed, it remains unclear whether the role of vertebrate *Pou5f1* in SC pluripotency was inherited from the ancestral paralog (see discussion in Gold et al., 2014). Our POU evolutionary tree shows a basal polytomy that prevents identifying the POU5 ancestor (Figure 4A). However, our tree is in agreement with a previous wide evolutionary analysis of the entire POU family (including POU1) in all Metazoa (Gold et al., 2014). In that study, a polytomous clade including POU5, POU3 and POU2 classes was identified, and the POU4 class was found as the paralog that diverged after the POU6+POU1 basal clade. Relying on the ancestral state reconstruction and on the taxonomic distribution of POU2 and POU5, Gold et al. (2014) hypothesized that a *Pou3*-like gene was the last common ancestor of the POU5/3/2 polytomous cluster, in which POU2 and POU5 classes represent bilaterian-specific and vertebrate-specific duplications, respectively. On this basis, and given the goal of our study, we investigated the expression pattern in *B. schlosseri* of both *Pou3*, as representative of the *Pou3*-like ancestor of *Pou5f1*, and *Pou2*, as the other closest paralog of *Pou5f1*.

Remarkably, while POU2 and POU4 are present in all analysed ascidians, *Pou3* was found only in ascidians of two Stolidobranchia families (*i.e.*, Styelidae and Pyuridae; Supplementary Table 1 and Figure 4A) and seems to be absent even in the two widely studied *Ciona* species with highly curated genomic sequence annotations. This leads us to hypothesize a *Pou3* loss in most ascidian groups, although the long branch/fast evolution of the ascidian *Pou3* clade (Figure 4A) could also explain the failure to identify these genes. As summarized in Figure 1D, we found that *B. schlosseri Pou3* is expressed in the endostyle, even in the adult phase, especially in the anterior-most part, close to the previously identified SC niche (Rinkevich et al., 2013). Moreover, *Pou3* is expressed in the few pluripotent cells of the early 2B and cSCs in their niches. This expression pattern highlights a likely role of *B. schlosseri Pou3* in undifferentiated cells, as also recently evidenced in *Botrylloides diegensis/leachii* (Viard et al., 2019; Kassmer et al., 2020). Overall, our and previous results support a role in SC pluripotency for the *Pou3*-like ancestor that gave rise to the vertebrate *Pou5f1,* here represented by the *Pou3* of ascidians. Finally, *Pou3* was found expressed in the differentiating heart, gut and gonads, and in the developing nervous system (see above).

As for *Pou2,* it is expressed exclusively in the immature previtellogenic oocytes, both in gonads and circulating in the colonial vascular system. Remarkably, this expression pattern significantly differs from those of vertebrates and invertebrates, where *Pou2* is expressed in many more territories. For example, *Pou2* is involved in neurogenesis in cephalopods (together with *Pou3*) (Wollesen et al., 2014), in the establishment of the dorsal/ventral axis in echinoderms (Range and Lepage, 2011) and in immunity in vertebrates (Strubin et al., 1995; Karnowski et al., 2012). Notably, this is the first report of *Pou2* expression in tunicates.

Overall, these results point to an expression pattern of the vertebrate *Pou5f1* more similar to that of *B. schlosseri Pou3* rather than *Pou2*, thus supporting the Gold’s and coll. (2014) hypothesis of a *Pou3*-like gene as ancestor of *Pou5f1*.

To summarize, we identified in the model species *B. schlosseri* the orthologs of vertebrate YFs through deep evolutionary analyses. Then, we found that *Myc*, *SoxB1* and *Pou3* are individually expressed in several differentiating territories, consistently with the reported function of these genes in chordate embryogenesis. Moreover, we found their expression in cells with features of cSCs exhibiting the same morphological and morphometric features. These cells are located close to the endostyle and in cell islands, and according to our analyses, should coexpress at least *Pou3* and *Myc*. Further studies, such as a deep analysis of niche dynamic and double *in situ* hybridization experiments, are needed to understand the actual co-expression of these factors in cSCs, which could in turn suggest a shared ancestral pluripotency program in chordates.

## Data Availability

The datasets presented in this study can be found in online repositories. The names of the repository and accession numbers can be found below: GenBank, OL828248-OL828252.
