# Supplementary material for: Yamanaka Factors in the Budding Tunicate Botryllus schlosseri Show a Shared Spatio-Temporal Expression Pattern in Chordates
Source: Front Cell Dev Biol. 2022 Mar 7;10:782722. doi: 10.3389/fcell.2022.782722 (PMC8948423; doi:10.3389/fcell.2022.782722)
Supplement: Supplementary file 4 [file DataSheet6.PDF]

Yamanaka Factors in the budding tunicate *Botryllus schlosseri* show a shared spatio-temporal expression pattern in chordates

Virginia Vanni[1‡], Marika Salonna[2‡], Fabio Gasparini[1], Margherita Martini[1], Chiara Anselmi[3,4], Carmela Gissi[2, 5,6,§]*, Lucia Manni[1§]*

[1]Department of Biology, University of Padova, Padova, Italy

[2]Department of Biosciences, Biotechnologies and Biopharmaceutics, University of Bari "Aldo Moro", Bari, Italy

[3]Stanford University, Hopkins Marine Station, Pacific Grove, CA 93950, USA

[4]Institute for Stem Cell Biology and Regenerative Medicine, Stanford University School of

Medicine, Stanford, CA 94305, USA

[5]IBIOM, Institute of Biomembranes, Bioenergetics and Molecular Biotechnologies, Consiglio Nazionale delle Ricerche, Bari, Italy

[6]CoNISMa, Consorzio Nazionale Interuniversitario per le Scienze del Mare, Roma, Italy

SUPPLEMENTARY FILE 6

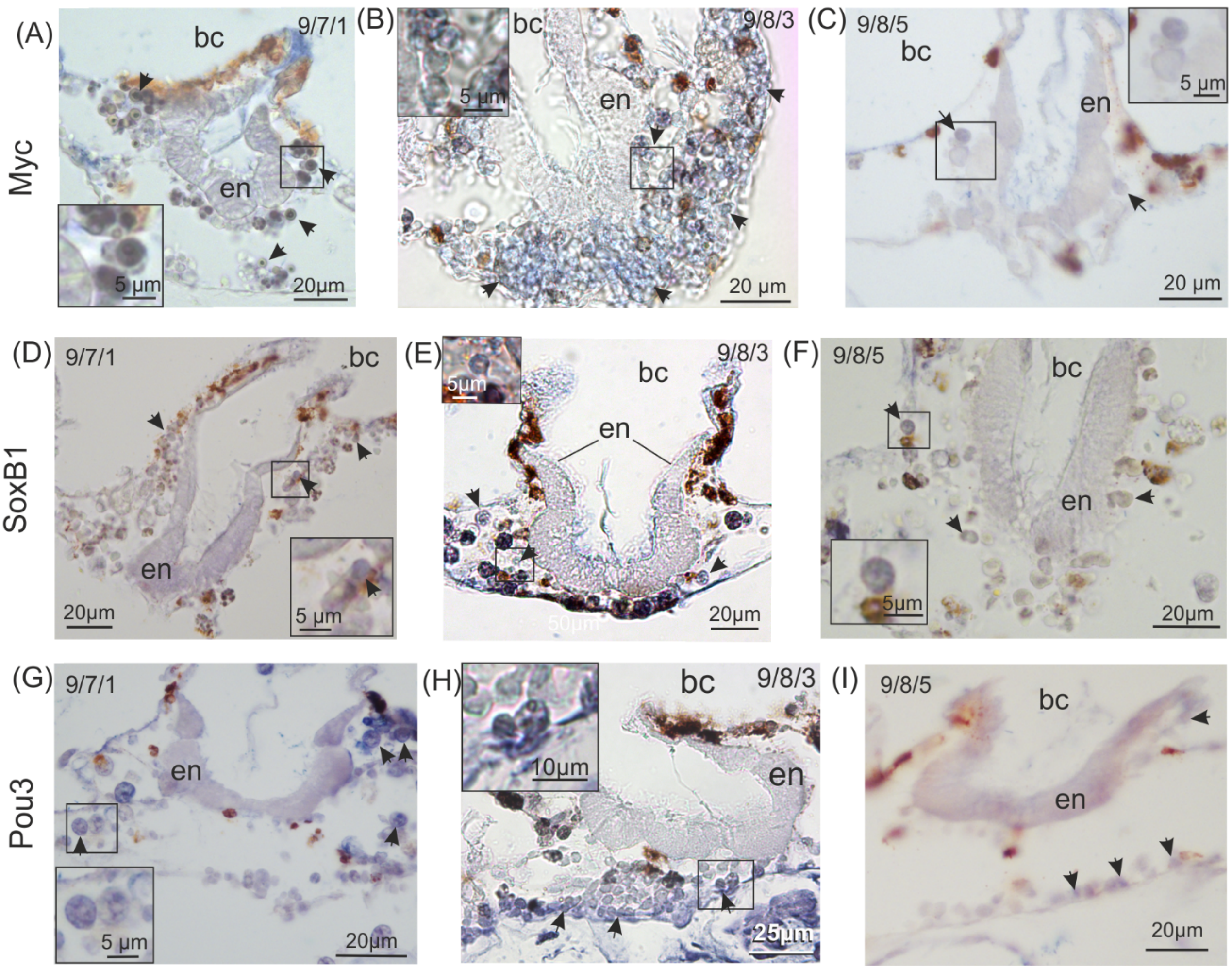

Figure 1 - Details of the endostyle niche in adult individuals in early- (9/7/1), mid- (9/8/3), and late- cycle (9/8/5), showing positive candidate SCs (labelled in blue, highlighted by black arrows) for the expression of *Myc*, *SoxB1,* and *Pou3*. *Pou2* was excluded as expressed only in the female germline.

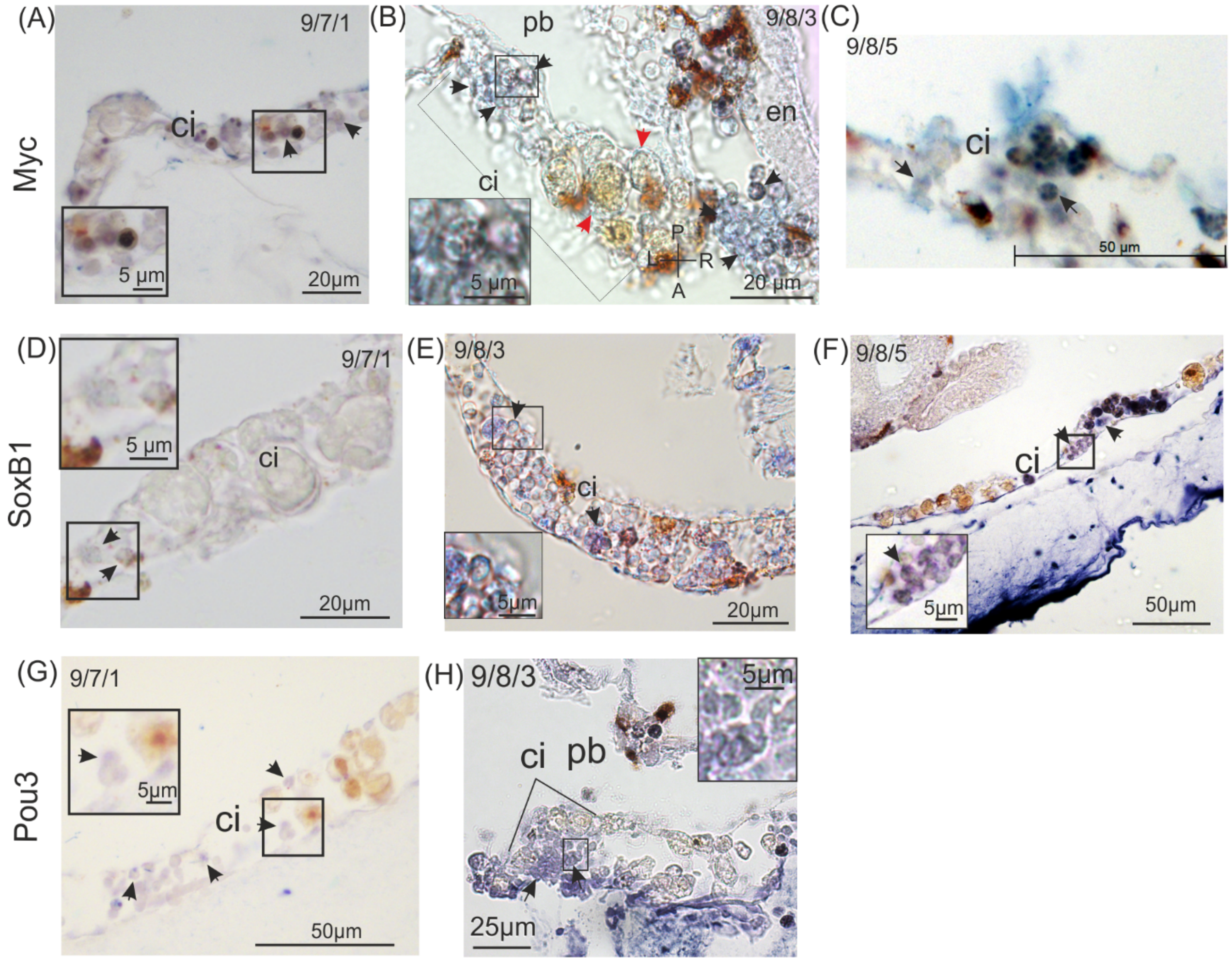

Figure 2 - Details of cell islands in adult individuals in early- (9/7/1), mid- (9/8/3), and late-cycle (9/8/5) of blastogenesis, showing positive candidate SCs (labelled in blue, highlighted by black arrows) for the expression of *Myc*, *SoxB1,* and *Pou3*. Cell islands were not found in late-cycle, as expected (Rinkevich et al., 2013). *Pou2* was excluded as expressed only in the female germline. Red arrows in (B): non-labelled phagocytes.

gene_stage	myc_971	myc_983	myc_985	soxB1_971	soxB1_983	soxB1_985	pou3_971	pou3_983	pou3_985
**cell 1**	4,50	4,29	5,96	6,58	5,18	4,03	5,99	4,25	6,65
**cell 2**	3,55	5,33	7,20	4,17	6,12	4,27	6,87	5,38	4,56
**cell 3**	5,00	4,18	5,50	5,18	4,92	4,59	4,29	5,56	4,79
**cell 4**	5,25	4,26	5,65	4,52	4,48	6,94	6,52	6,23	4,29
**cell 5**	4,76	4,29	5,14	6,63	3,89	6,62	4,69	3,34	4,61
**cell 6**	3,98	4,46	4,82	4,82	5,33	6,36	6,47	5,63	6,44
**cell 7**	4,03	3,84	5,92	6,68	5,37	4,92	4,36	4,22	5,43
**cell 8**	4,18	4,55	5,11	4,34	6,28	6,78	5,01	3,84	4,54
**cell 9**	5,27	4,14	6,39	4,23	4,83	5,93	6,06	5,79	5,58
**cell 10**	4,38	5,81	5,91	5,31	3,05	6,64	4,04	3,29	7,31
**Mean**	4,49	4,51	5,76	5,25	4,94	5,71	5,43	4,75	5,42
**Standard deviation**	0,58	0,60	0,69	1,03	0,97	1,13	1,06	1,08	1,05

	Myc	SoxB1	Pou3		
**Mean per gene**	4,92	5,30	5,20	**Mean Total**	5,14
**St_dev per gene**	0,85	1,06	1,08	**St_dev Total**	0,97

Table 1 - Diameter (in micron) of 10 hemoblasts (candidate SCs) found positive for each gene in niches in adult samples fixed at early- (9/7/1), mid- (9/8/3) and late-cycle (9/8/5) of blastogenesis. Mean and standard deviation reported for each column and, in the boxes at the bottom, for each gene and for the total population considered.

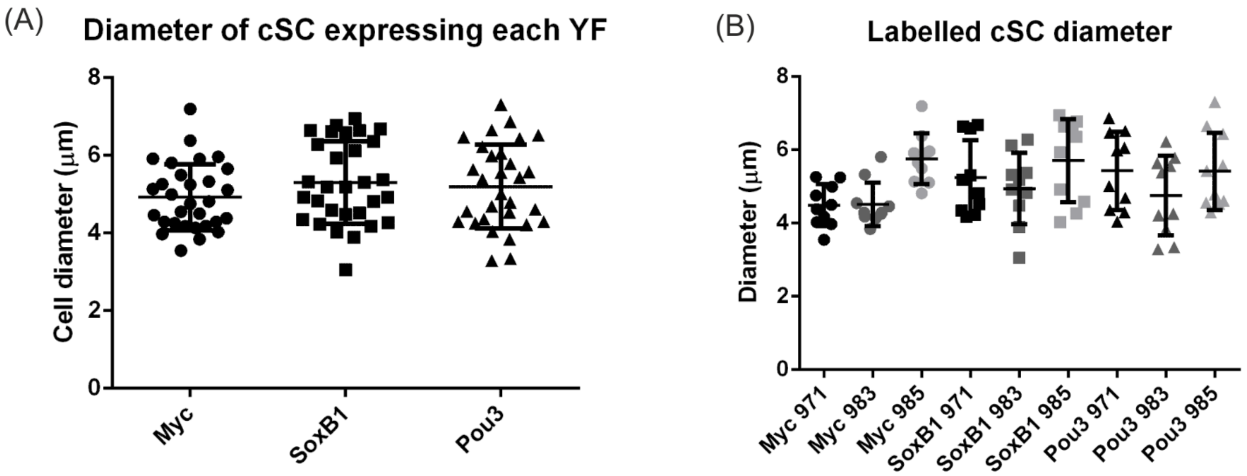

Figure 3 - Graphs based on data of Table 1 showing the diameter of candidate SCs found positive for each gene in (A) and for each gene and stage in (B). ANOVA test shows no significant differences among the groups of diameters of candidate SCs expressing each individual gene.

	myc_971		myc_983		myc_985		soxB1_971		soxB1_983		soxB1_985		pou3_971		pou3_983		pou3_985	
	labelled	total	labelled	total	labelled	total	labelled	total	labelled	total	labelled	total	labelled	total	labelled	total	labelled	total
EN 1	11	16	51	61	2	3	7	26	3	10	8	25	6	10	23	31	6	13
EN 2	19	26	11	16	3	5	10	66	4	9	4	12	7	9	9	17	4	7
EN 3	10	17	36	49	5	6	3	42	7	38	3	25	8	10	30	37	3	9
CI 1	5	8	8	11	3	5	0	5	6	20	3	25	6	12	15	21	na	na
CI 2	8	12	7	12	3	6	3	11	2	7	8	13	13	17	10	11	na	na
CI 3	5	11	10	13	na	na	2	13	3	25	7	12	5	7	7	9	na	na

Table 2 - Number of labelled and total hemoblasts found in three different sections of endostyle niches and cell islands (EN and CI respectively) in adult samples fixed at early- (9/7/1), mid- (9/8/3) and late-cycle (9/8/5) of blastogenesis.

	myc_971	myc_983	myc_985	soxB1_971	soxB1_983	soxB1_985	pou3_971	pou3_983	pou3_985
**EN 1**	68,75	83,61	66,67	26,92	30,00	32,00	60,00	74,19	46,15
**EN 2**	73,08	68,75	60,00	15,15	44,44	33,33	77,78	52,94	57,14
**EN 3**	58,82	73,47	83,33	7,14	18,42	12,00	80,00	81,08	33,33
**CI 1**	62,5	72,73	60,00	0,00	30,00	12,00	50,00	71,43	na
**CI 2**	66,67	58,33	50,00	27,27	28,57	61,54	76,47	90,91	na
**CI 3**	45,45	76,92	na	15,38	12,00	58,33	71,43	77,78	na
**Mean**	62,55	72,30	64,00	15,31	27,24	34,87	69,28	74,72	45,54
**Standard deviation**	9,72	8,47	12,34	10,76	11,16	21,53	11,84	12,63	11,92

	Myc	SoxB1	Pou3
**Mean per gene**	66,42	25,81	66,71
**Standard deviation**	10,51	16,60	15,93

Table 3 - Percentage of labelled hemoblasts found in three different endostyle niches and cell islands (EN and CI respectively) in adult samples fixed at early (9/7/1), mid (9/8/3) and late (9/8/5) phases of the blastogenetic cycle (data from Table 2). Mean and standard deviation are reported for each column and, in the box at the bottom, for each gene.

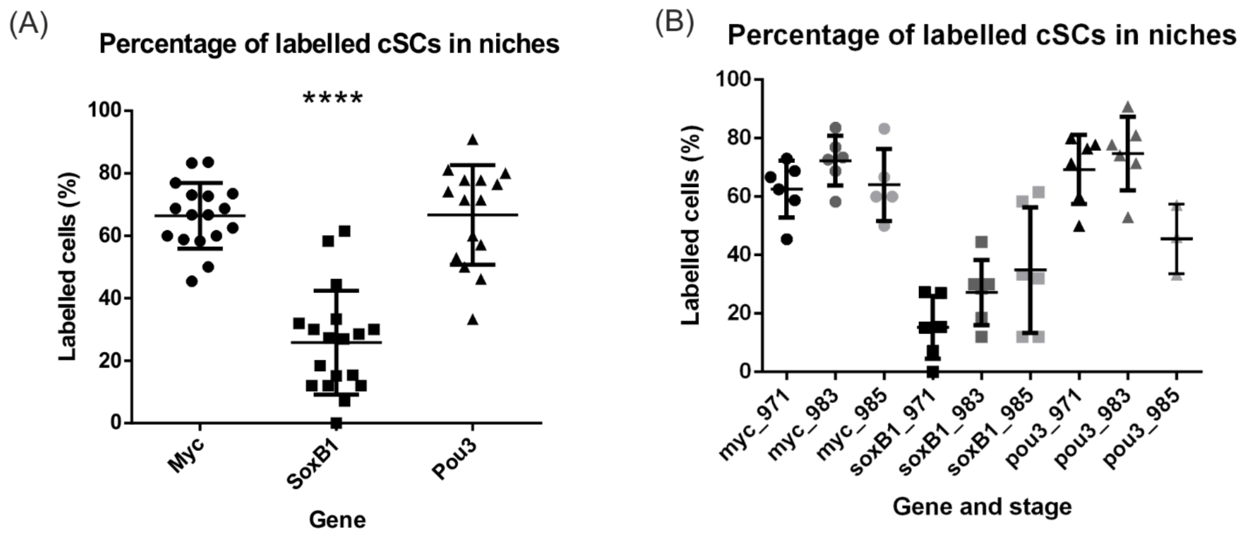

Figure 4 - Graphs based on data of Table 3 showing the percentage of candidate SCs found positive for each gene in (A) and for each gene and stage in (B) in endostyle and cell island niches in adult individuals. ANOVA test, and Tukey test for multiple comparisons show that the percentage of candidate SCs expressing *SoxB1* are significantly less numerous than those expressing *Myc* and *Pou3* (****<0.001 in A). The latter genes are expressed in the same percentage of cells.
