# Supplementary figures and images for: Yamanaka Factors in the Budding Tunicate Botryllus schlosseri Show a Shared Spatio-Temporal Expression Pattern in Chordates
Source: Front Cell Dev Biol. 2022 Mar 7;10:782722. doi: 10.3389/fcell.2022.782722 (PMC8948423; doi:10.3389/fcell.2022.782722)

Supplementary  
Fig 2A

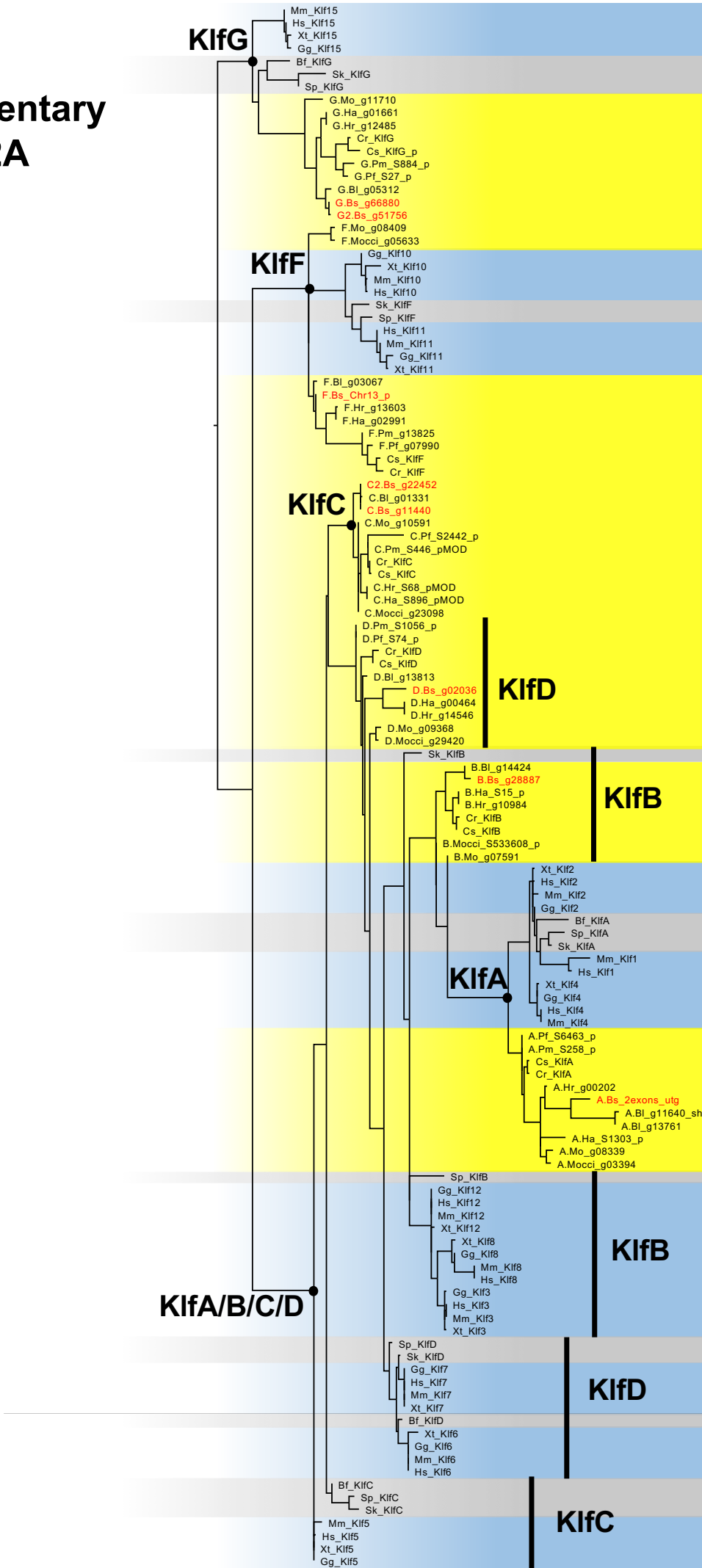

Supplementary  
Fig 2B

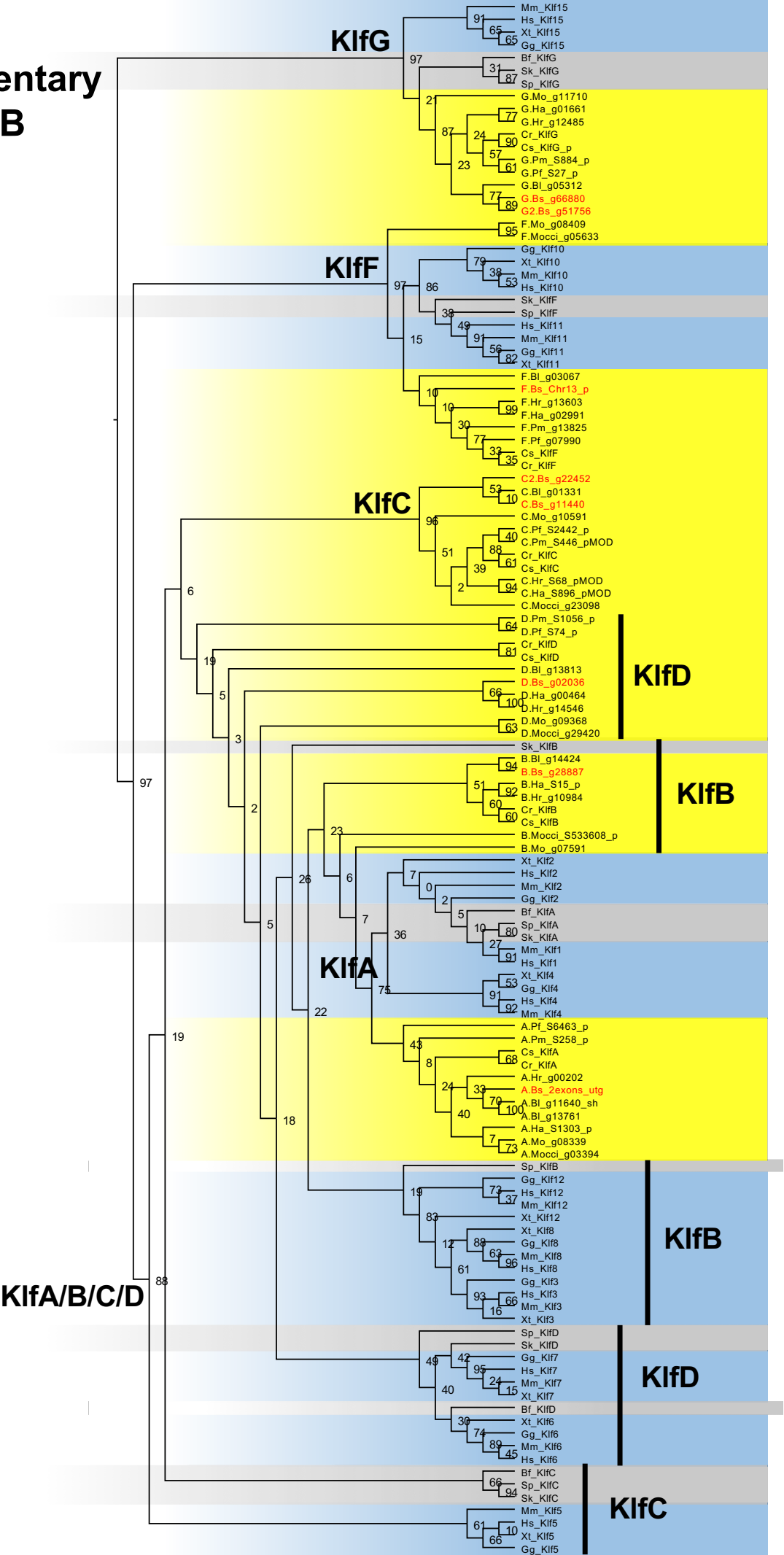

Supplement: Supplementary file 6 [file Image2.PDF]

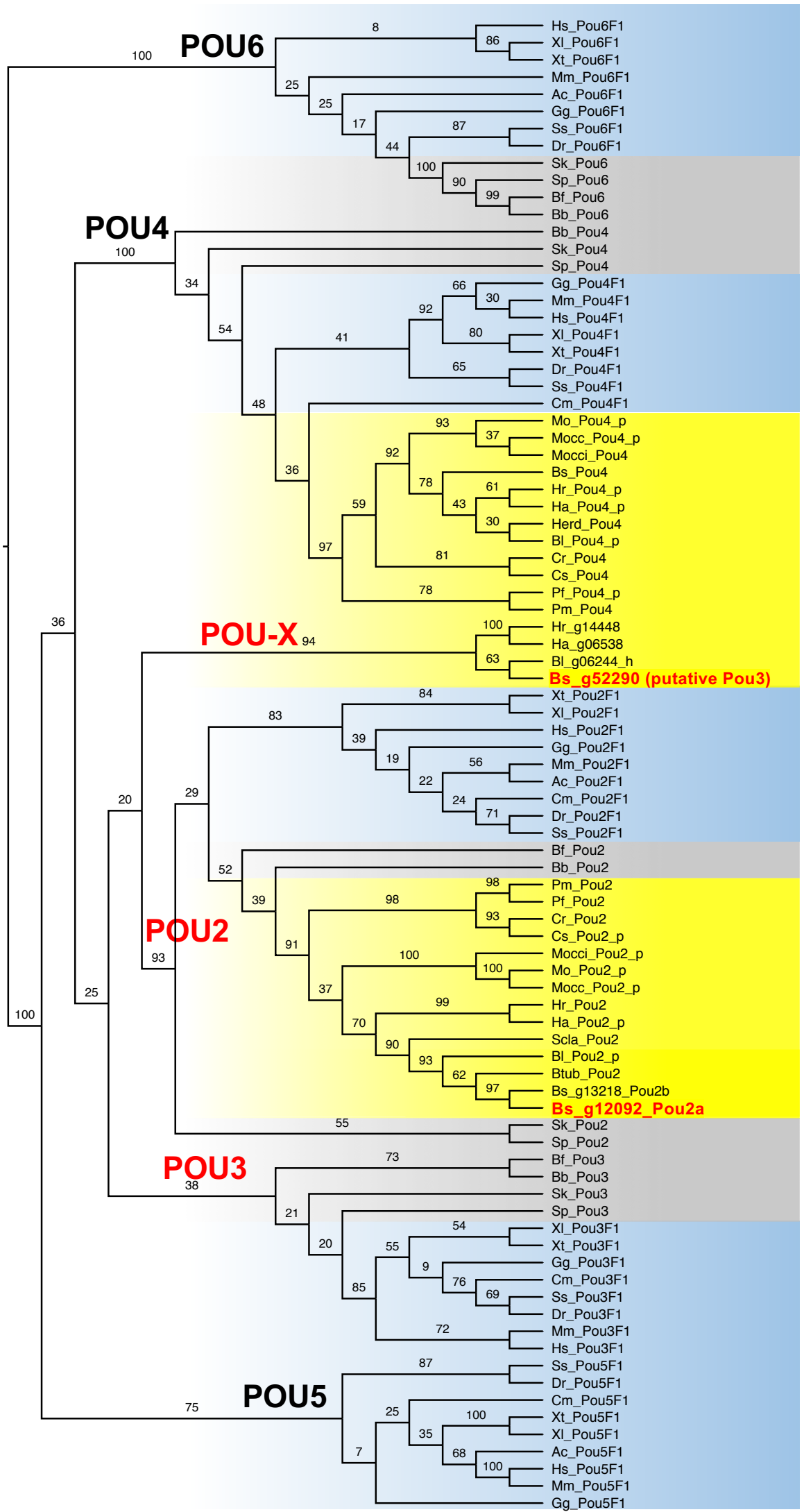

Supplement: Supplementary file 13 [file Image1.PDF]
