# Supplementary material for: Yamanaka Factors in the Budding Tunicate Botryllus schlosseri Show a Shared Spatio-Temporal Expression Pattern in Chordates
Source: Front Cell Dev Biol. 2022 Mar 7;10:782722. doi: 10.3389/fcell.2022.782722 (PMC8948423; doi:10.3389/fcell.2022.782722)
Supplement: Supplementary file 8 [file DataSheet1.PDF]

Yamanaka Factors in the budding tunicate *Botryllus schlosseri* show a shared spatio-temporal expression pattern in chordates

Virginia Vanni[1ǂ], Marika Salonna[2ǂ], Fabio Gasparini[1], Margherita Martini[1], Chiara Anselmi[3,4], Carmela Gissi[2, 5,6,§*], Lucia Manni[1§*]

[1]Department of Biology, University of Padova, Padova, Italy

[2]Department of Biosciences, Biotechnologies and Biopharmaceutics, University of Bari "Aldo Moro", Bari, Italy

[3]Stanford University, Hopkins Marine Station, Pacific Grove, CA 93950, USA

[4]Institute for Stem Cell Biology and Regenerative Medicine, Stanford University School of Medicine, Stanford, CA 94305, USA

[5]IBIOM, Institute of Biomembranes, Bioenergetics and Molecular Biotechnologies, Consiglio Nazionale delle Ricerche, Bari, Italy

[6]CoNISMa, Consorzio Nazionale Interuniversitario per le Scienze del Mare, Roma, Italy

SUPPLEMENTARY FILE 1

METHODS

YFs identification and evolution in Chordates

To reconstruct the evolution of the YF gene families in chordates, we searched for orthologs and paralogs of human YFs in the genomic and transcript databases of *B. schlosseri* as well as the genomic databases of 10 additional ascidians of the Stolidobranchia and Phlebobranchia orders. The only available genome of an Aplousobranchia, belonging to *Didemnum vexillum*, was not analysed since about 84.78 % of this assembly consists of contigs < 1 kb in length (Velandia et al., 2016), making almost impossible the annotation of large and complex genes such as those for the Yamanaka factors. The YFs of 14 deuterostome representatives (including vertebrates, cephalochordates, hemichordates and echinoderms) and other ascidian species were identified in the nr-nucleotide database of NCBI (https://www.ncbi.nlm.nih.gov/nucleotide/). Species lists and queried databases are reported in **Supplementary Table 1**. These genes, selected through tBLASTn (Altschul et al., 1990), were first multi-aligned to identify deuterostome evolutionarily conserved regions guiding the annotation corrections and the *ex-novo* annotations in ascidians. The GeneWise tool, which predicts gene sequences and structures using similar protein sequences (https://www.ebi.ac.uk/Tools/psa/genewise/) (Birney et al., 2004), was applied to the ascidian genomic contigs encoding for YFs identified by tBLASTn, using as an input the YFs protein sequences of congeneric or closely related species. When available, RNAseq tracks present in ANISEED (Brozovic et al. 2018) were also considered in gene re-annotations. The Untranslated regions (UTR) of the *B. schlosseri* genes were annotated using available transcript sequences (Campagna et al., 2016; Prünster et al., 2019) and checking related RNAseq tracks in ANISEED (Brozovic et al., 2018). For each YF family, **Supplementary Tables 2-5** present the final analysed datasets, and **Supplementary Files 2-5** report the sequences annotated *ex novo* or re-annotated. All multi-alignments of the protein-coding sequences (CDS) were performed with the web-server TranslatorX (Abascal et al., 2010), choosing ClustalW (Thompson et al., 1994) or Mafft (Katoh et al., 2005) as alignment software and manually optimized with Seaview v4.6.4 (Gouy et al., 2010).

Maximum likelihood (ML) phylogenetic trees were reconstructed at the amino acid level using the online software PHYML v3.0 (http://www.atgc-montpellier.fr/phyml-sms/) (Guindon and Gascuel, 2003), which also includes the automatic model selection algorithm SMS (Smart Model Selection). The best-fit substitution model was selected using the Akaike Information Criterion (AIC). Bootstrap values, indicating node reliability, were based on 100 replicates. Only for POU sequences, additional analyses with 1000 replicates were performed using PHYML as well as RAxML-NG v1.0.1 (Kozlov et al., 2019), available at the CIPRES Science Gateway (Miller et al., 2010; www.phylo.org). A posteriori bootstrap convergence tests were then carried out with RAxML-NG v1.0.1, setting the autoMRE criterion and a cutoff threshold of 0.03 as bootstopping options.

RNA extraction, YFs cDNA cloning and sequencing

In order to validate the *B. schlosseri* predictions of *SoxB1*, *Myc*, *Pou2* and *Pou3* and to develop probes for ISH experiments, total RNA was extracted from single colonies of *B. schlosseri* following the protocol described by Campagna et al. (2016). Specific primers were designed for each predicted gene using the software Primer QuestTool-IDT (https://eu.idtdna.com). Primer sequences, PCR details and amplicons mapping on the genomic sequences are reported in the next section "**cDNA amplification protocol**". First strand cDNA synthesis was performed using mixed random primers and anchored oligo(dT)15 primers, according to the Improm II Reverse Transcription System (Promega) manual, followed by amplifications with the Biotaq DNA Polymerase (Bioline) or the DreamTaq polymerase (Thermo Fisher Scientific). Amplicons were then cloned with the TOPO TA cloning kit (Thermo Fisher scientific) and sequenced with the Sanger method at BMR Genomics (Italy) or Microsynth AG (Switzerland). Some amplicons were also purified with the DNA Clean&Concentrator kit (Zymo research) and directly sequenced for gene prediction confirmation. Sequence quality check, assembly, and comparisons to the predicted genes were carried out with Geneious ver. 5.5.7.2 (Kearse et al., 2012).

cDNA amplification protocol in *B. schlosseri*

Primer sequences and primer pair combinations used in cDNA amplifications are described in Table A1.

cDNA amplifications of *Myc, SoxB1*, and *Pou3* were carried out with the Biotaq DNA Polymerase (Bioline) in a 25 µl reaction volume containing: 1X NH4 reaction buffer, 8mM final concentration of $MgCl_2$, 0.2 mM of each dNTP, 0.4 µM of each primer and 2.5 units of Biotaq DNA polymerase. Amplification conditions consisted of an initial denaturation for 2 min at 94°C, then 35 amplification cycles divided as follows: denaturation for 30 sec at 94°C, annealing for 30 sec at 63°C, extension for 1 min or 1 min 30 sec (depending on the length of the amplicon) at 72 °C, a final elongation step of 8 min at 72°C.

PCRs of *Pou2* were performed with the DreamTaq polymerase (Thermo Fisher Scientific) in a final volume of 25 µl containing: 1X reaction buffer with 2 mM final concentration of $MgCl_2$, 0.2 mM of each dNTP, 0.4 µM of each of the two primers, and 1.25 units of DreamTaq polymerase. The amplification conditions were: an initial denaturation for 3 min at 95°C, then 30 amplification cycles (denaturation for 30 sec at 95°C; annealing for 30 sec at 52-60°C depending on the primer pair; extension for 1 min or 1 min 30 sec at 72 °C, depending on the length of the amplicon) followed by a final elongation step of 7 min at 72°C.

Amplicon sequences were assembled and deposited in the GenBank database with accession numbers OL828248-OL828252.

B. schlosseri colonies rearing

Colonies of *B. schlosseri* were collected in the Lagoon of Venice and reared at the Department of Biology (University of Padova) in standard laboratory condition (Sabbadin, 1955, 1960). Colonies were observed daily and fixed at 6 developmental phases as defined by the staging method developed by Sabbadin (1955) (Figure 1A of the main text).

Histology and Transmission Electron Microscopy (TEM) protocols

For histology, colonies were anaesthetized with MS222 for 5–10 minutes and fixed in 4% paraformaldehyde in phosphate-buffered saline (pH 7.4). Specimens were then dehydrated with ethanol at crescent concentrations and embedded in Paraplast. Serial sections 7 µm thick were cut and counterstained with haematoxylin–eosin.

For TEM, after treatment with anesthetic, selected fragments of colonies were fixed in 1.7% glutaraldehyde buffered with 0.2M sodium cacodylate plus 1.6% NaCl, pH 7.4. Then, they were washed in buffer, post-fixed in 1% OsO4 in 0.2 M cacodylate buffer, dehydrated and embedded in epoxy resin (Sigma-Aldrich). Ultrathin sections (80 nm thick) were stained with uranyl acetate and lead citrate to provide contrast. Photomicrographs were taken with a FEI Tecnai G12 electron microscope operating at 100 kV. Images were captured with a Veleta (Olympus Soft Imaging System) digital camera.

In situ hybridization

Specific *SoxB1*, *Myc*, *Pou2* and *Pou3* sequenced clones were used as templates for the synthesis of sense and antisense RNA probes labelled with digoxigenin-linked nucleotides, using T7, T3 and SP6 RNA polymerases (Promega) according to the supplier's protocol.

The probes used in the *in situ* hybridization experiments are described in red in Table A1 and graphically mapped on the relative gene/genomic sequences in Figures A1 to A4.

Whole-mount ISHs were carried out as previously described (Franchi and Ballarin, 2014) using at least three samples per stage. Probes were purified with mini-Quick Spin Columns (Roche). Firstly, specimens were fixed overnight in freshly prepared MOPS buffer with 4% paraformaldehyde (MOPS 0.5 M, NaCl 2.5M, MgSO4 5 mM, EGTA 10 mM). Samples were dehydrated using graded PBST (PBS/0.1% Tween-20)/Methanol (50%, 70% and 100%) then washed in xylene and rehydrated with graded PBST/Methanol (100%, 90%, 80%, 50%, 30%). Specimens were permeabilized with 10 µg/ml Proteinase K in PBST, washed in PBST and post-fixed in a 4% paraformaldehyde plus 0.2% glutaraldehyde solution in PBST. They were incubated in the hybridisation mix (Amresco) for 1 h at 63°C and then overnight with 1µg/ml DIG-labelled riboprobes. They were washed with washing solution #1 (W1:SSC 5X pH 4.5, SDS 10%, formamide 50%) and washing solution #2 (W2:NaCl 0.5M, Tris-HCl 10 mM pH 7.5, Tween-20 0.1%) as follows: twice with W1 (30 min at 60°C) , twice with W1+W2 (10 min at 60°C), twice with W2 (5 min at 37°C), once with W2+RNase 20µg/ml (30 min at 37°C), once with W2 (5 min at 37°C), twice with W1+W2 (10 min at 60°C), once with W1 (10 min at 60°C), once with W1+TBST (NaCl 137 mM, KCl 2,7 mM, Tris-HCl 25 mM, Tween-20 0.1%) (10 min at 60°C), twice with TBST (10 min at 60°C). Specimens subsequently were: i) treated with TBST for 5 min three times at room temperature (RT), ii) incubated in a blocking solution (1% milk powder in TBST) 4 h at 4°C, iii) incubated in blocking solution with TBST and an anti-digoxigenin antibody conjugated with alkaline phosphatase (Ab-antiDIG) for 12-16h at 4°C. Washes in TBST were carried out on ice as follows: 10 min, 20 min (twice) and 60 min (three times). Staining was performed with NTM (Tris-HCl 2M pH 9.5, NaCl 2M, MgCl2 2M) for 3 min, three times at RT. NTM + NBT-BCIP (Sigma), for 24 hours at room temperature, away from light. Then samples were incubated in NTM for 10 minutes at RT and after in Tris-EDTA, for 10 minutes. Washes with water for 5 minutes were carried out twice, followed by on ice graded dehydration with Ethanol in PBST (50%, 70%, 90% and 100%) and a final step with xylene (30 min at RT and 30 at 60°C). Finally,

specimens were embedded in Paraplast and sectioned. As a result, hybridized portions of the specimens could be detected with images acquired using a Leica 5000B microscope. Control experiments were treated with sense probes and gave no labelling (see Figure A5). Some sections (three per gene in colonies at early-, mid-, and late-cycle) were used for the morphometric and cell quantification studies of candidate SCs. Data were statistically analysed using GraphPad PRISM 6 Software.

B. schlosseri YF gene re-annotation and ISH probes

Myc

Figure A1 shows the original ANISEED annotation of the *B. schlosseri Myc* gene (Boschl.CG.Botznik2013.chr11.g41847), corresponding to a partial CDS, together with our re-annotation of the complete CDS (Bs_Myc_p), and the cDNA amplicons sequenced to confirm our re-annotation. The presence of a long intron of 13272 bp was first *in silico* predicted and then confirmed by RT-PCR and cDNA sequencing with the Myc_F2/Myc_R2 primer pair (the forward primer Myc_F2 falls at the exon1-exon2 junction; see Table A1 and Figure A1). Similarly, the 3'UTR was first inferred from the transcript TCONS_00134812 (Campagna et al. 2016) and from RNAseq data (data not shown from A. Voskoboynik and C. Anselmi), and then most of this 3'UTR was confirmed by cDNA sequencing (see amplicons Myc_F3/Myc_R3 and Myc_F4/Myc_R4 in Figure A1). The used ISH probe (in red in Figure A1) corresponds to a region encompassing the end of the CDS and a portion of the 3'UTR.

SoxB1 and SoxB2

Figure A2 shows the original ANISEED annotation of the *B. schlosseri SoxB1* gene (Boschl.CG.Botznik2013.chr13.g61081) together with our re-annotation (Bs_SoxB1_p). The re-annotation of the first exon of *B. schlosseri SoxB1* was initially guided by the observation of the high conservation of the protein N-terminus in all ascidians, and then validated by analyses of available transcripts (Campagna et al. 2016) and RNAseq data (data not shown from A. Voskoboynik and C. Anselmi), as well as by cDNA amplification and sequencing with the primer pair SoxB1_F1/SoxB1_R1 (Figure A2). The 5' and 3' UTRs of *B. schlosseri SoxB1* were inferred from the transcript g61081.t1.1 (Campagna et al. 2016) and then a portion of both these UTRs was validated by cDNA amplification and sequencing (Figure A2). The used ISH probe (in red in Figure A2) consists of the end of the CDS and the beginning of the 3'UTR.

The re-annotation of *B. schlosseri SoxB2* is described in Figure A6. Two putative *SoxB2* genes (Boschl.CG.Botznik2013.chrUn.g33885 and Boschl.CG.Botznik2013.chrUn.g49627) were found in ANISEED, both mapping on an "unknown chromosome". They are almost identical in sequence but have a different length and lack the C-terminal protein region, highly conserved in all other analysed ascidians. Our re-annotation includes the identification of this highly conserved C-terminal region. Although it was not experimentally validated, we believe that our re-annotation is highly reliable since: a) it has the same 4-exons gene structure found in all other ascidians; b) the identified C-terminus shows a very high similarity among ascidians, and c) the encoded protein is 93% similar (89% identical) to that of the closest related species *Botrylloides leachii*. However, it has to be noted that our *SoxB2* re-annotation assumes genome assembly errors, causing not only a *SoxB2* abnormal gene structure but also an artefactual gene duplication. Indeed, our *SoxB2* gene maps entirely on region 347M, but the last exon is 40 Mbp apart and in opposite orientation compared to the other three exons (see mapping region 347M in Figure A6). Moreover, a second incomplete gene copy, truncated at the C-terminus and with 99% nucleotide identity to the previous one, was found about 85 Mbp apart (see mapping region 221M in Figure A6).

Pou genes

Figure A3 and A4 show the annotation of the *B. schlosseri Pou2a* and *Pou-X* (the putative *Pou3*) genes, respectively, together with the mapping on the genomic sequence of the analysed cDNA amplicons and the used ISH probes. Both these genes correspond to the original ANISEED gene models, and their annotation was confirmed by our gene family evolutionary analyses and RT-PCR/cDNA sequencing.

The *Pou2* ascidian genes show a conserved intron/exon gene structure. Indeed, most ascidian *Pou2* genes consist of 10 coding exons, with the exceptions of 11 coding exons in the two analysed *Halocynthia* species and in *Styela clava,* and the exceptions of 9 coding exons in the two analysed *Phallusia* species (see **Supplementary Table 4**). However, the presence of a first coding exon characterized by a low evolutionary conservation and a short size (4-29 bp) could have hampered the identification of the true first exon and therefore of the correct intron/exon gene structure in the above-mentioned species.

The *Pou4* coding sequence was obtained from a transcript published by Prünster et al., (2019) (Bot_trin2_112211_c0_seq1). As shown in Figure A7, this transcript is not annotated in ANISEED (Brozovic et al. 2018; https://www.aniseed.cnrs.fr) but it maps:

- with 99% identity on chr13;
- with 97% identity on two regions of chrUn located about 137Mbp apart (Figure A7).

On both mapping chromosomes, a gene structure consisting of 9 exons can be identified. While the mapping on chr13 does not show anomalies, the mapping on chrUn is characterized by the first exon and the beginning of the second exon being anomalously located downstream of exons 3-9 and at a large distance of about 137Mb. So we hypothesize an error in genome assembly, causing an additional and artifactual *Pou4* gene copy on chrUn.

Noteworthy, *Pou4* ascidian genes show a quite conserved gene structure. Indeed, almost all *Pou4* genes consist of 8-10 coding exons (see **Supplementary Table 4**). This mild variability could be explained by the existence of alternative splicing isoforms (see Gene ID: 497238 of *Ciona intestinalis* - https://www.ncbi.nlm.nih.gov/gene/497238) as well as by the presence of a very short final coding exon, which makes it difficult to predict. Indeed, the experimentally validated final exon of *Ciona Pou4* encodes only for 4 amino acids (evolutionary conserved, as shown by our alignment), the stop codon, and the entire 3'UTR (Candiani et al., 2005). We found a final coding exon of only 4-5 amino acids in several ascidian *Pou4* (see **Supplementary Table 4**). Only the *Pou4* genes of the closely related species *Molgula oculata* and *Molgula occulta* have significantly different gene structures, with 4 and 3 coding exons respectively, although the *M. occulta Pou4* annotation could also be partial as it lacks the short final coding exon described above.

Table A1. Primer pairs used in RT-PCR of the analysed YFs genes in *B. schlosseri*. Amplicons used as probes in ISH are reported in red. CDS: coding sequence; UTR: untranslated region. * Size calculated from the 3'end of the primers. $ Different combinations of the above-listed Pou2 primers.

Primer	Sequence (5'->3')	Tm (°C)	Located in coding exon ...	Amplicon (bp) *	Transcript region
**Myc**					
Myc_F2	TCAGAGGAAGAAATCGATGTGG	62	boundary 1°-2°	674	CDS
Myc_R2	AGCGTCAAGAAGGAAGTACG	62	2°		
Myc_F3	ACGCGTATTCGTATCCAACC	62	2°	771	CDS + 3'UTR
Myc_R3	AAACCGTCTTTCTCTGATACTGG	62	2°		
Myc_F4	TGTCACCTCTGTCTTCTCTACC	63	2°	520	CDS + 3'UTR
Myc_R4	AACTGTCATCATTCCTCCATCC	62	2°		
**SoxB1**					
SoxB1_F1	CACTGTACTGACCGGAAACG	62	1°	194	5'UTR + CDS
SoxB1_R1	GGCTCTGTGCTGAATAAGTAGG	62	2°		
SoxB1_F3	CGTACACAGTCTCTCCTATACCC	63	3°	464	CDS + 3'UTR
SoxB1_R3	CGTACCTTGTTGTGGTAAGTCC	63	3°		
SoxB1_F4	GCCAGAGGATGAACACATTCC	63	3°	284	CDS + 3'UTR
SoxB1_R4	GCGAAATTGTAACGAGCAAACC	63	3°		
**Pou3**					
Pou3_F1	ACGAACATTACCGACCATATCC	62	1°	472	CDS
Pou3_R1	GATAATCCACTTCTCCGTAGCC	62	1°		
Pou3_F2	CGAAACTATTATCCAGCGTCTACC	63	1°	547	CDS

Primer	Sequence (5’->3’)	Tm (°C)	Located in coding exon ...	Amplicon (bp) *	Transcript region
Pou3_R2	TTCTTGCGTCGTAGGTTTGG	62	1°		
Pou3_F3	CTTACACCAACGATGCAAACG	62	1°	507	CDS
Pou3_R3	TAGTGGTTTGGCTGAATACGG	62	1°		
**Pou2**					
Pou2_F1	TCAAGTGAACCAACTTCTGACC	63	1°	240	CDS
Pou2_R1	GCTTGAAATCCTTTGCGAACC	62	5°		
Pou2_F2	TGGAATGCATCCTGGAATATCG	62	3°	444	CDS
Pou2_R2	TTTCTTCTGTGGTCGGTTTAGG	62	9°		
Pou2_F3	CGGAAGATCTCAACATGGATCG	63	9°	257	CDS
Pou2_R3	GTTGAATGATACCGGGTCTGG	63	10°		
Pou2_F1	TCAAGTGAACCAACTTCTGACC	63	1°	593 $	CDS
Pou2_R2	TTTCTTCTGTGGTCGGTTTAGG	62	9°		
Pou2_F2	TGGAATGCATCCTGGAATATCG	62	3°	757 $	CDS
Pou2_R3	GTTGAATGATACCGGGTCTGG	63	10°		
Pou2_F1	TCAAGTGAACCAACTTCTGACC	63	1°	906 $	CDS
Pou2_R3	GTTGAATGATACCGGGTCTGG	63	10°		

Figure A1. Map of the Bs_Myc_p gene, together with the relative cDNA amplicons and the ISH probe, on the genomic sequence Boschl_2013 available in ANISEED

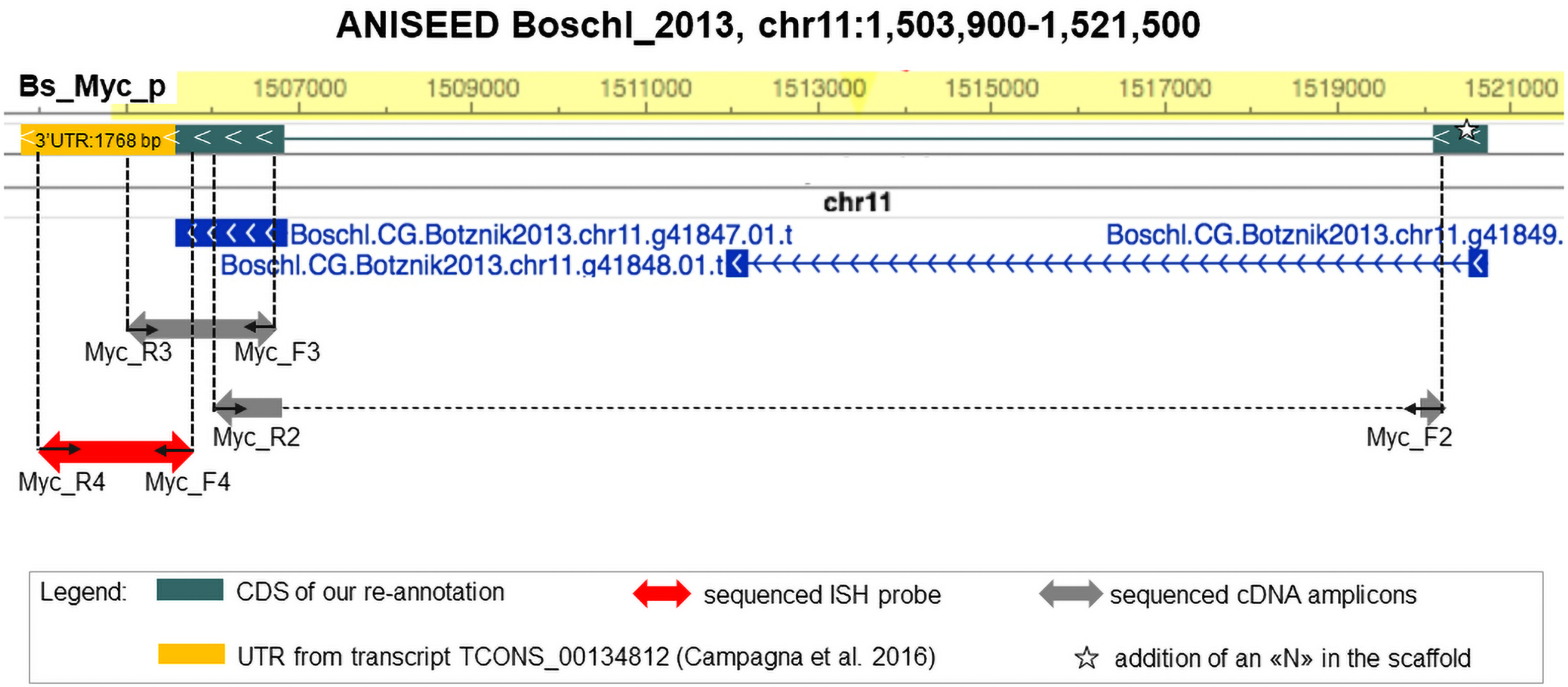

Figure A2. Map of the Bs_SoxB1_p gene, together with the relative cDNA amplicons and the ISH probe, on the genomic sequence Boschl_2013 available in ANISEED.

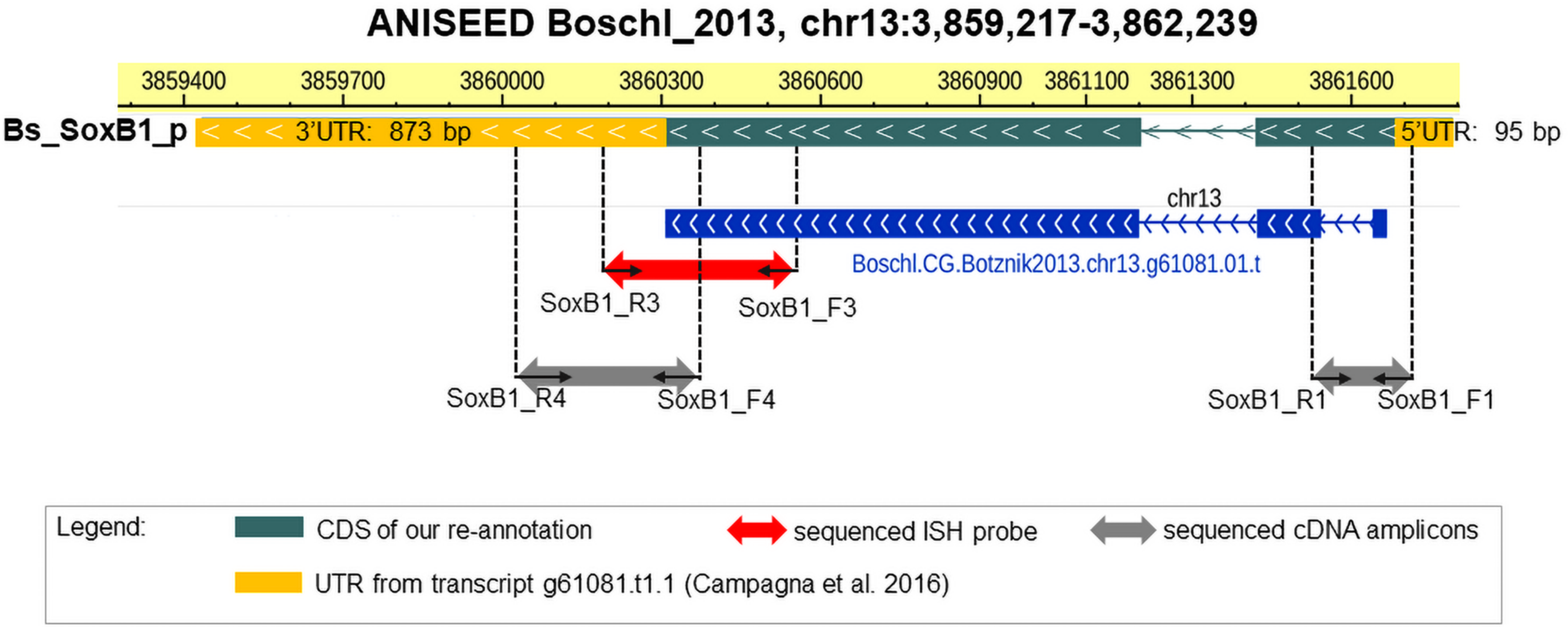

Figure A3. Map of the Bs_g12092_Pou2a gene, together with the relative cDNA amplicons and the ISH probe, on the genomic sequence Boschl_2013 available in ANISEED.

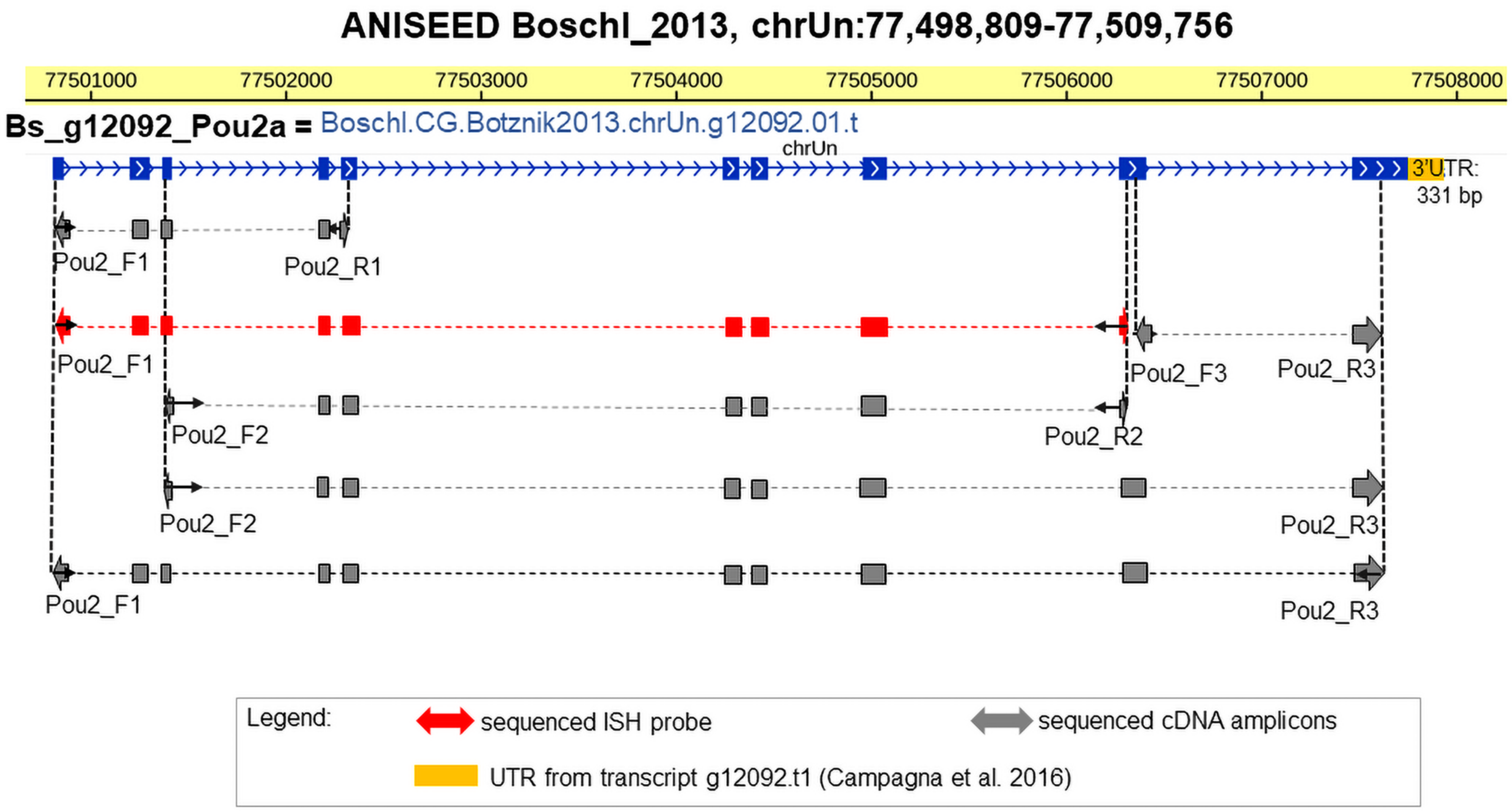

Figure A4. Map of the putative *B. schlosseri* Pou3 gene, Bs_g52290, together with the relative cDNA amplicons and the ISH probe, on the genomic sequence Boschl_2013 available in ANISEED.

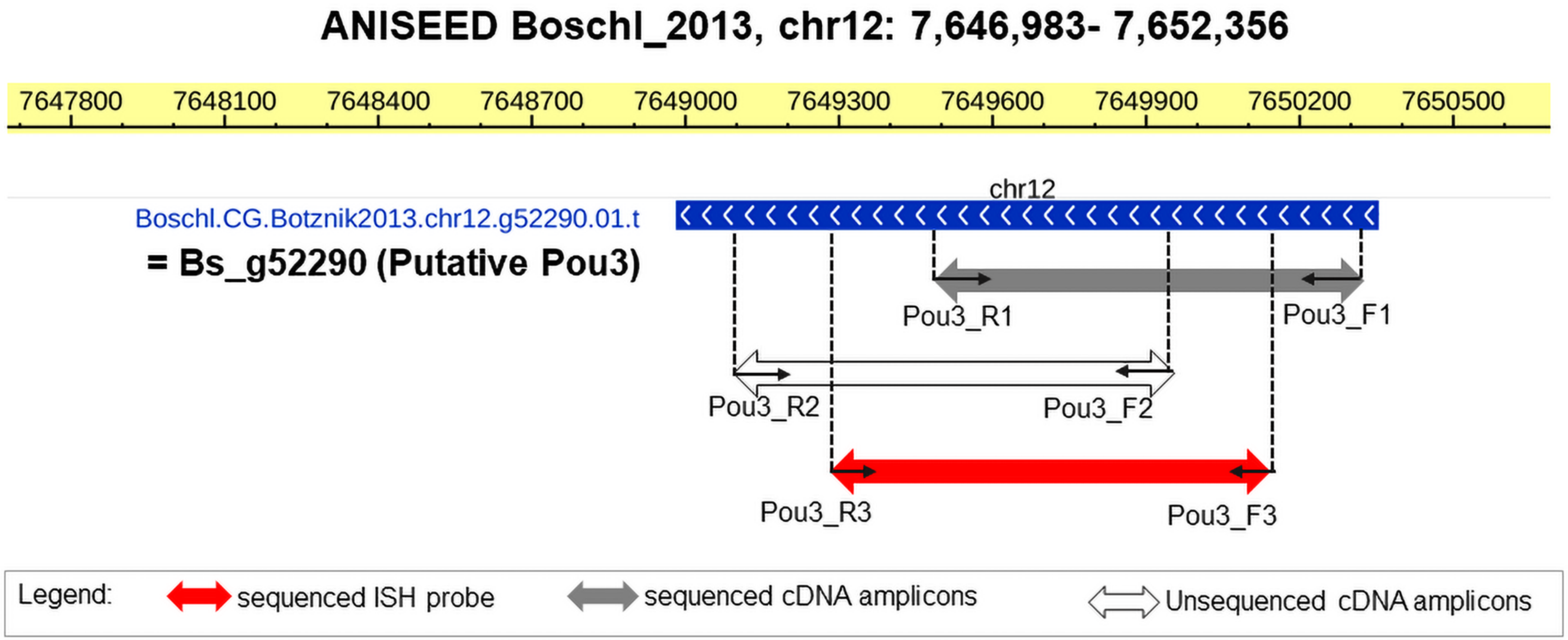

Figure A5. Control experiments treated with the sense probes. Left: *Myc* sense probe. Center: *SoxB1* sense probe. Right: *Pou3* sense probe. Tissues are not labelled. Tunic shows the characteristic aspecific blue labelling (see at Middle and Right). Scale bar: 50 µm

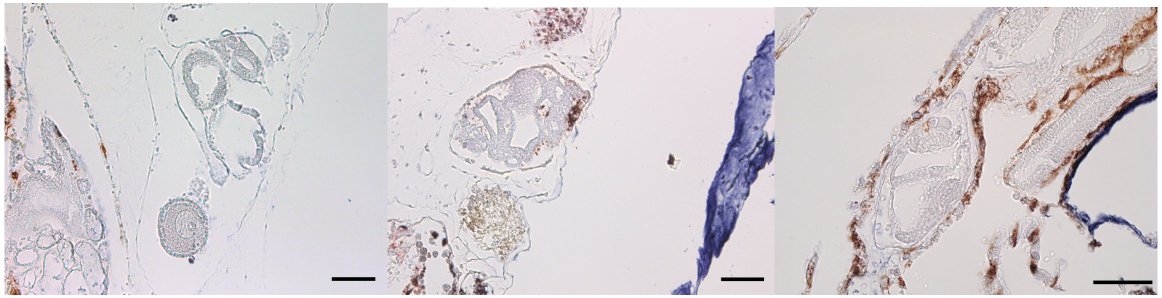

Figure A6. Map of the Bs_SoxB2_p gene on the genomic sequence Boschl_2013 available in ANISEED.

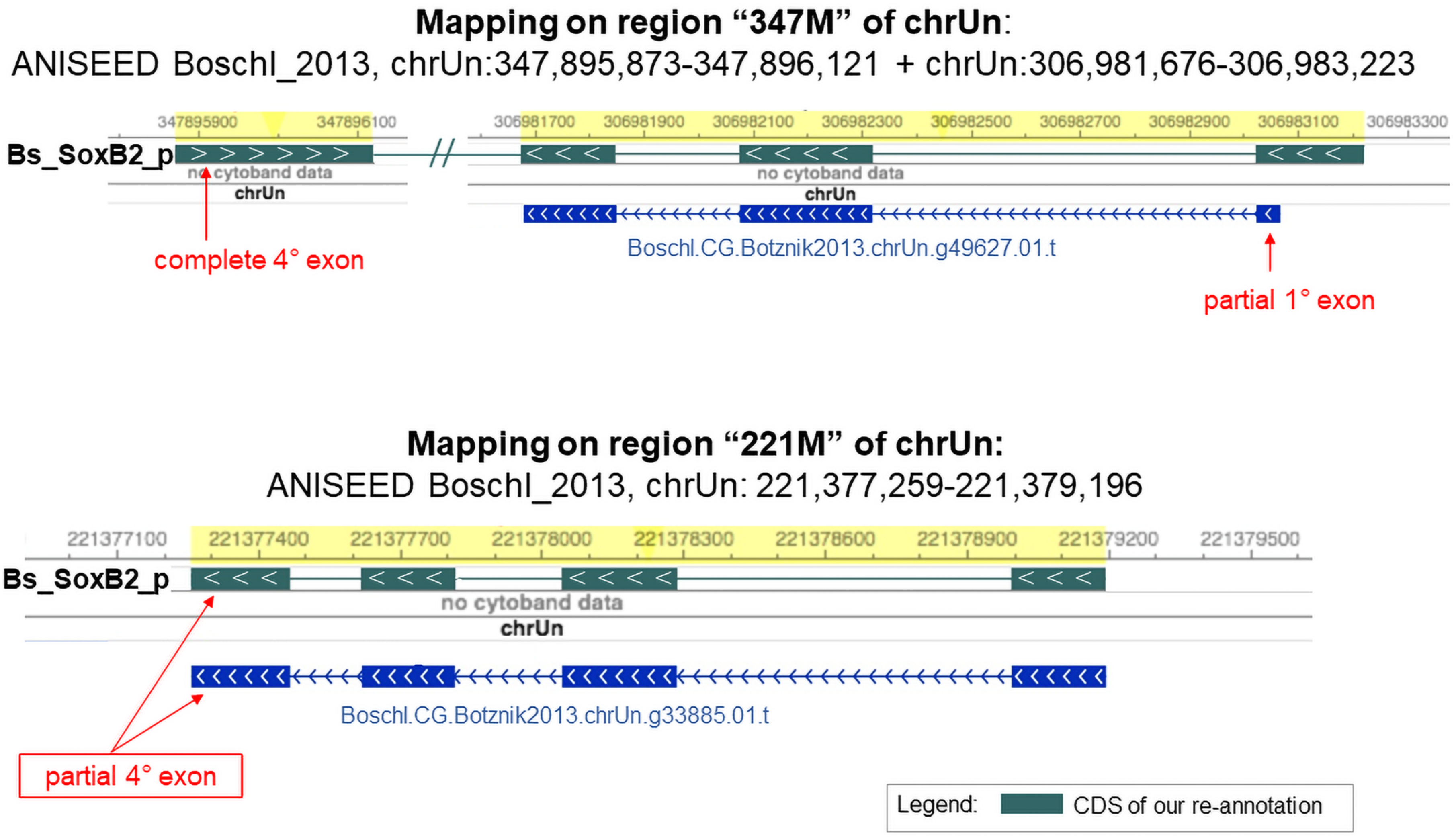

Figure A7. Map of the Bs_Pou4 gene on the genomic sequence Boschl_2013 available in ANISEED.

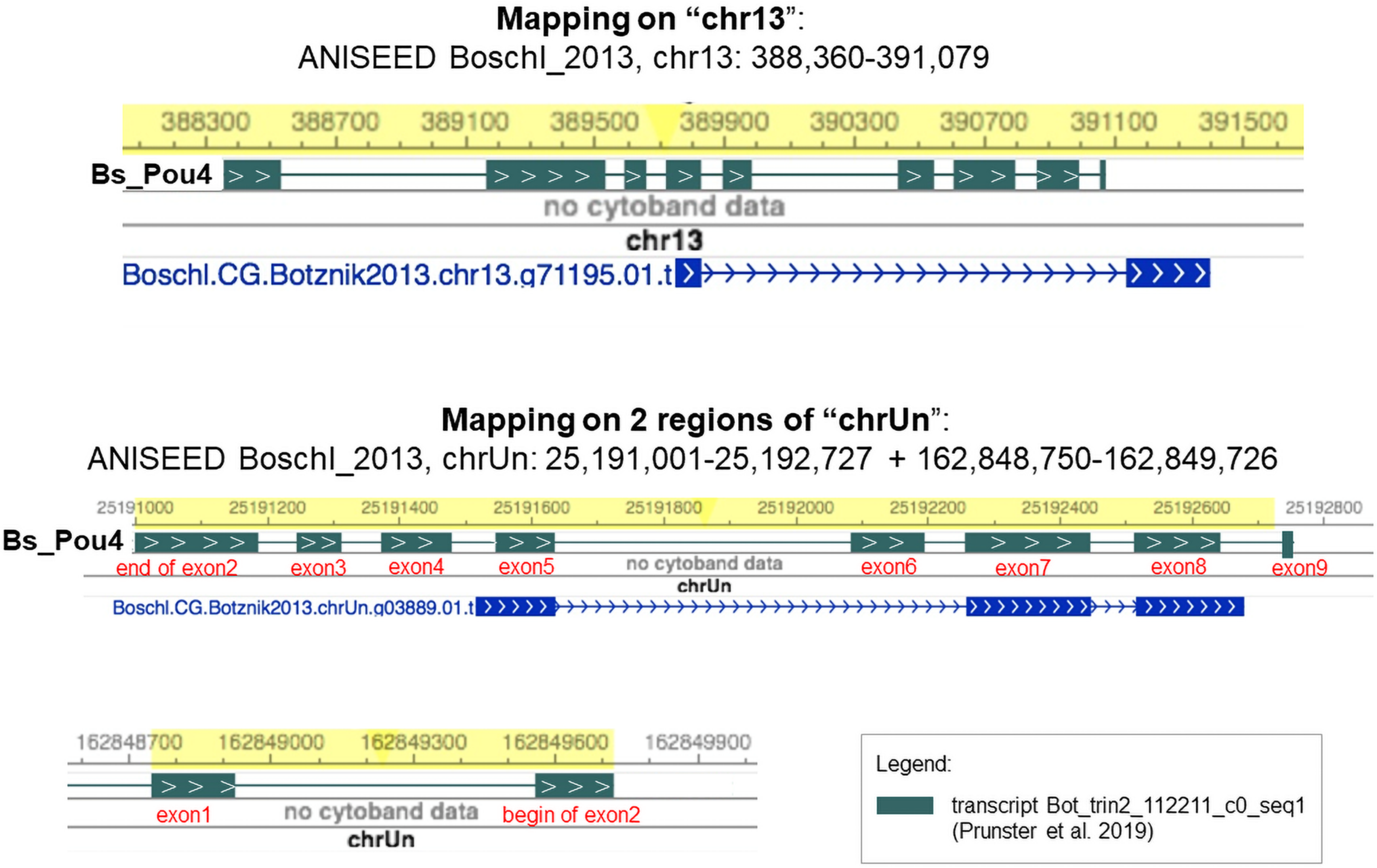

REFERENCES

Abascal, F., Zardoya, R., and Telford, M. J. (2010). TranslatorX: multiple alignment of nucleotide sequences guided by amino acid translations. *Nucleic Acids Res*. 38, W7-13. doi:10.1093/nar/gkq291.

Altschul, S. F., Gish, W., Miller, W., Myers, E. W., and Lipman, D. J. (1990). Basic local alignment search tool. *J. Mol. B*iol. 215, 403–410. doi:10.1016/S0022-2836(05)80360-2.

Birney, E., Clamp, M., & Durbin, R. (2004). GeneWise and Genomewise. *Genome Res*. 14(5), 988–995. doi:10.1101/gr.1865504

Brozovic, M., Dantec, C., Dardaillon, J., Dauga, D., Faure, E., Gineste, M., Louis, A., Naville, M., Nitta, K. R., Piette, J., et al. (2018). ANISEED 2017: extending the integrated ascidian database to the exploration and evolutionary comparison of genome-scale datasets. *Nucleic Acids Res.* 46, D718–D725. doi:10.1093/nar/gkx1108.

Campagna, D., Gasparini, F., Franchi, N., Vitulo, N., Ballin, F., Manni, L., Valle, G., and Ballarin, L. (2016). Transcriptome dynamics in the asexual cycle of the chordate *Botryllus schlosseri. BMC Genomics* 17, 275. doi:10.1186/s12864-016-2598-1.

Candiani, S., Castagnola, P., Oliveri, D., and Pestarino, M. (2002). Cloning and developmental expression of AmphiBrn1/2/4, a POU III gene in amphioxus. *Mech. Dev.* 116, 231–234. doi:10.1016/s0925-4773(02)00146-6.

Gouy, M., Guindon, S., and Gascuel, O. (2010). SeaView version 4: A multiplatform graphical user interface for sequence alignment and phylogenetic tree building. *Mol. Biol. Evol.* 27, 221–224. doi:10.1093/molbev/msp259.

Franchi, N., & Ballarin, L. (2014). Preliminary characterization of complement in a colonial tunicate: C3, Bf and inhibition of C3 opsonic activity by compstatin. *Dev. Comp. Immunol.* 46(2), 430–438. doi:10.1016/j.dci.2014.05.014

Guindon, S., and Gascuel, O. (2003). A simple, fast, and accurate algorithm to estimate large phylogenies by maximum likelihood. *Syst. Biol.* 52, 696–704. doi:10.1080/10635150390235520.

Katoh, K., Kuma, K., Toh, H., and Miyata, T. (2005). MAFFT version 5: improvement in accuracy of multiple sequence alignment. *Nucleic Acids Res.* 33, 511–518. doi:10.1093/nar/gki198.

Kearse, M., Moir, R., Wilson, A., Stones-Havas, S., Cheung, M. *et al.,* (2012) Geneious Basic: an integrated and extendable desktop software platform for the organization and analysis of sequence data. *Bioinformatics,* 28, 1647-9. doi: 10.1093/bioinformatics/bts199

Kozlov, A. M., Darriba, D., Flouri, T., Morel, B., and Stamatakis, A. (2019). RAxML-NG: a fast, scalable and user-friendly tool for maximum likelihood phylogenetic inference. *Bioinformatics* 35, 4453–4455. doi:10.1093/bioinformatics/btz305.

Miller, M., Pfeiffer, W. T., and Schwartz, T. (2010). Creating the CIPRES Science Gateway for Inference of Large Phylogenetic Trees. *2010 Gateway Computing Environments Workshop (GCE),* pp. 1-8. doi:10.1109/GCE.2010.5676129.

Prünster, M. M., Ricci, L., Brown, F. D., and Tiozzo, S. (2019). *De novo* neurogenesis in a budding chordate: Co-option of larval anteroposterior patterning genes in a transitory neurogenic organ. *Dev. Biol.* 448, 342–352. doi:10.1016/j.ydbio.2018.10.009.

Sabbadin, A. (1960). Nuove Ricerche Sull' Inversione Sperimentale Situs Viscerum *Botryllus schlosseri* Sabbadin. *Estratto dall'Archivio di Oceanografia e Limnologia* 11.

Sabbadin, A. (1955). Osservazioni sullo sviluppo, l'accrescimento e la riproduzione di *Botryllus schlosseri* (Pallas), in condizioni di laboratorio. *Bolletino di Zoologia* 22, 243–263. doi:10.1080/11250005509439204.

Thompson, J. D., Higgins, D. G., and Gibson, T. J. (1994). CLUSTAL W: improving the sensitivity of progressive multiple sequence alignment through sequence weighting, position-specific gap

penalties and weight matrix choice. *Nucleic Acids Res*. 22, 4673–4680. doi:10.1093/nar/22.22.4673.

Velandia-Huerto, C. A., Gittenberger, A. A., Brown, F. D., Stadler, P. F., and Bermúdez-Santana, C. I. (2016). Automated detection of ncRNAs in the draft genome sequence of a colonial tunicate: the carpet sea squirt *Didemnum vexillum*. *BMC Genomics* 17, 691. doi:10.1186/s12864-016-2934-5.
